# Risk of Guillain–Barré syndrome after vaccination against human papillomavirus: a systematic review and meta-analysis, 1 January 2000 to 4 April 2020

**DOI:** 10.2807/1560-7917.ES.2022.27.4.2001619

**Published:** 2022-01-27

**Authors:** T Sonia Boender, Barbara Bartmeyer, Louise Coole, Ole Wichmann, Thomas Harder

**Affiliations:** 1 Department of Infectious Disease Epidemiology, Robert Koch Institute, Berlin, Germany; 2 Postgraduate Training for Applied Epidemiology (PAE), Robert Koch Institute, Berlin, Germany; 3 European Programme for Intervention Epidemiology Training (EPIET), European Centre for Disease Prevention and Control (ECDC), Stockholm, Sweden; 4 Field Service, UK Health Security Agency, Leeds, United Kingdom

**Keywords:** vaccination, Papillomaviridae, Guillain-Barre syndrome, Systematic Review

## Abstract

**Background:**

Guillain–Barré syndrome (GBS) is a rare autoimmune disease that can follow viral infections and has in a few cases been linked to vaccinations. Pre-licensure clinical trials did not observe an association between human papillomavirus (HPV) vaccination and GBS, a post-marketing study from 2017 reported an increased relative risk.

**Aim:**

We assessed the risk of GBS after HPV vaccination through a systematic literature review and meta-analysis.

**Methods:**

We searched Embase, MEDLINE and Cochrane for studies reporting on the risk of GBS after HPV vaccination in individuals aged ≥ 9 years, published between 1 January 2000 and 4 April 2020, excluding studies without a comparator group. Seven studies reporting relative effect sizes were pooled using random-effects meta-analysis. We assessed quality of evidence using the GRADE approach. Study protocol was registered (PROSPERO No. #CRD42019123533).

**Results:**

Of 602 identified records, we included 25 studies. Based on over 10 million reports, cases of GBS were rare. In 22 studies no increased risk was observed, while in three studies a signal of increased risk of GBS after HPV vaccination was identified. Meta-analysis yielded a pooled random-effects ratio of 1.21 (95% CI: 0.60–2.43); I^2^ = 72% (95% CI: 36–88). This translates to a number needed to harm of one million to be vaccinated to generate one GBS case. Quality of evidence was very low.

**Conclusions:**

The absolute and relative risk of GBS after HPV vaccination is very low and lacks statistical significance. This is reassuring for the already implemented vaccination programmes and should be used in respective communication activities.

## Background

More than 10 years after the licensure of the first human papillomavirus (HPV) vaccines, a growing body of evidence supports the large-scale implementation of HPV immunisation programmes. Clinical trials and post-marketing observational studies have shown consistent efficacy, effectiveness and safety of the available HPV vaccines: (i) the bivalent Cervarix, (targeting HPV types 16 and 18, GlaxoSmithKline Biologicals, Rixensart, Belgium); (ii) the 4-valent recombinant Gardasil (targeting HPV types 6, 11, 16, and 18, MSD VACCINS, Lyon, France) and; (iii) the 9-valent Gardasil 9 (targeting HPV types 6, 11, 16, 18, 31, 33, 45, 52 and 58, MSD VACCINS) [1,2]. Vaccination against HPV reduces the prevalence and incidence of cervical intraepithelial neoplasia grade 2 or 3 or worse (CIN2/3 or worse) among girls and women and anogenital warts diagnoses among girls, women, boys and men [3]. Since 2007, HPV vaccination programmes have been implemented in most European countries, usually targeting females. In recent years, several countries have extended their recommendation to a gender-neutral programme [4]. Notably, herd effects have been measured in countries with high HPV vaccination coverage [3,5].

The future public health impact of HPV vaccination on HPV-associated disease will rely on the vaccination coverage achieved. While the expansion of vaccination programmes is encouraging and uptake is increasing, overall HPV vaccination rates remain low and below national targets in a number of countries [6,7]. Suboptimal vaccination coverage is often driven by vaccine hesitancy, which in turn is often related to public debates and fear of vaccine-induced side effects [8,9].

Among the possible risks associated with vaccination, Guillain–Barré syndrome (GBS) is one of the most serious. Guillain–Barré syndrome is a rare autoimmune disease where the body’s immune system damages nerve cells, causing muscle weakness and, in some cases, paralysis. Most people recover, however, some have lasting long-term weakness and GBS can be fatal. It occurs with a frequency of less than one case per 100,000 person-years in the age group relevant for HPV vaccination, i.e. those aged 10–19 years [10,11]. The causes of GBS are not yet fully understood, however, it often occurs after viral or bacterial infections and, in rare cases, after vaccination [12].

While pre-licensure clinical trials showed no association between HPV vaccination and subsequent occurrence of GBS, a French study from 2017 reported a more than threefold increased relative risk [13]. No evidence of an association between HPV vaccination and any autoimmune disorder has been found so far [9]. To the best of our knowledge, there is no systematic literature review investigating the potential association between the HPV vaccination and GBS specifically. We assessed the available evidence on the risk of GBS after HPV vaccination by including both randomised controlled trials (RCT) and post-marketing non-randomised studies.

## Methods

We registered our systematic literature review protocol at the International Prospective Register for Systematic Reviews (PROSPERO) under the registration number CRD42019123533 [14]. We report our results according to the Preferred Reporting Items for Systematic Reviews and Meta-Analyses (PRISMA) reporting guideline [15].

We included all studies reporting on individuals aged 9 years and older (i.e. the licenced age) who have been vaccinated against HPV with one of these vaccines: the bivalent Cervarix; (ii) the 4-valent recombinant Gardasil and; (iii) the 9-valent Gardasil 9. We included all possible vaccination schedules, including stopped schedules.

We did not restrict any study design, however, we excluded studies lacking a comparator group. and any type of control group would suffice, given the fact that there is one (e.g. placebo, no/other vaccination). We did not restrict our search based on language or geography.

The outcome of interest was GBS after HPV vaccination, as sub-defined by Brighton criteria [16] and all other non-Brighton criteria. According to the Brighton criteria, GBS includes acute inflammatory demyelinating polyradiculoneuropathy and acute motor axonal neuropathy. Other, non-Brighton criteria include Miller Fisher syndrome, which is a subtype of GBS characteristically consisting of the triad of ataxia, areflexia, ophthalmoplegia, acute motor and sensory axonal neuropathy and overlap syndromes between GBS and Miller Fisher syndrome.

We reviewed all literature reporting on the risk of occurrence of GBS after HPV vaccination, published between 1 January 2000 and 21 January 2019 and indexed in Embase, MEDLINE and the Cochrane Central Register of Controlled Trials. On 2 April 2020, we updated our search and included an additional search for publications in PubMed. We provide the full search strategy in the Supplement. Additionally, we used the snowballing approach to include additional studies by hand-searching the citation lists of included studies.

### Study selection and data collection

We uploaded all records to Covidence, a screening and data extraction tool for systematic reviews. Two reviewers (TSB and TH) independently included and excluded studies, using a stepwise approach based on title and abstract screening and a subsequent full-text screening.

Subsequently, one reviewer (TSB) extracted data from the included studies using a pre-defined data extraction sheet (the data extraction sheet is included in the Supplement). The second reviewer (TH) revised the data extractions against the original papers to identify potential errors. In case of disagreement, a final decision was made by consensus between the reviewers.

From the included studies, we extracted: (i) information on the study set-up (design, location, study period and follow-up time in person-years, inclusion and exclusion criteria); (ii) study population, (sex; age; number of people included in total, and by vaccinated/control group); (iii) intervention (type of vaccine used); (iv) control group; (v) potential co-interventions and; (vi) outcome (GBS definition; source of outcome reporting; incidence in the HPV-vaccinated and control groups).

When available, we also extracted the incidence rate (IR) and all reported measures of association, including the incidence rate ratio (IRR), relative risk (RR), odds ratio (OR), hazard ratio (HR) and potentially corrected confounding factors. Furthermore, we collected funding source and reported conflict of interest as risk of bias indicators.

### Assessment of risk of bias and quality of the body of evidence

Two reviewers (TSB and TH) independently assessed included studies for risk of bias. For RCT we used the revised Cochrane Collaboration’s tool (RoB 2.0) [17] and for non-randomised studies the Risk of Bias in Non-randomized Studies - of Interventions (ROBINS-I) tool was used [18]. The overall assessment of the quality of the body of evidence followed the Grading of Recommendations Assessment, Development, and Evaluation (GRADE) approach [19] in its most recent version adapted for use of ROBINS-I [20].

### Meta-analysis

All relative effect measures were pooled into one relative effect measure (ratio). Between-study heterogeneity in random-effects meta-analysis is reported through I^2^. Meta-analyses were conducted using the meta package in R version 3.6.3 (R Foundation for Statistical Computing, Vienna, Austria).

Pre-specified subgroup analyses were planned to explore the potential effect of study design, vaccine type (i.e. bivalent vaccine, 4-valent recombinant vaccine and 9-valent vaccine) and GBS case definition (Brighton vs non-Brighton) on the pooled effect estimate. When multiple studies were reporting on the same data source but with different subgroups of reporting timeframes, the most recent and/or most complete study was used for the primary analysis. Sensitivity analysis was performed by vaccine type and by outcome measurement to assess the robustness of the results of primary meta-analyses.

### Public health perspective

To assess the public health implications of our findings, we calculated the number needed to harm (NNH) to generate one additional case of GBS using GRADEprofiler version 3.6 (Informer Technologies, Los Angeles, United States (US)), based on the pooled findings of this study and the baseline risk for the age group of 10–19-year-olds (males: 0.97/100,000; females: 0.55/100,000; overall 0.75/100,000) [21]. For comparison, the number needed to vaccinate (NNV) was calculated in respect to the prevention of one case of cervical cancer [22].

## Results

**Figure 1:**
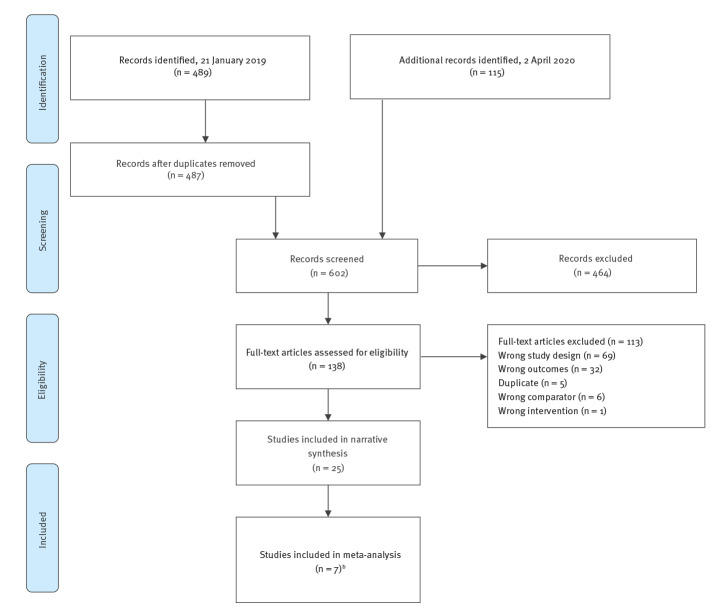
PRISMA flow diagram of studies that were screened to identify the risk of Guillain–Barré syndrome after vaccination against human papillomavirus, 1 January 2000–4 April 2020 (n = 602)^a^

In total, we identified and screened 602 citations and included 25 studies (Figure 1).

PRISMA: Preferred Reporting Items for Systematic Reviews and Meta-Analyses reporting guideline.

^a^ The full search strategy and list of the excluded full-text papers can be found in the Supplement.

^b^ Two studies published findings with a large overlap in data based on the VAERS dataset [34,35] with similar findings. The effect estimate by Geier et al. 2017 [35] was used in the primary analyses and Geier et al. 2015 [34] estimate in the sensitivity analysis. Therefore, the results of the meta-analysis show findings of six studies.

### Study characteristics

#### Population

The 25 included studies (Table 1) were conducted between 1999 and 2017. Twelve studies were conducted in Europe [13,23-33] and 12 studies were conducted in North America [34-45]. One study reported pooled RCT outcomes [46] without reporting the study location(s).

**Table 1:** Characteristics of the included studies reporting on the risk of Guillain–Barré syndrome after human papillomavirus vaccination, 1 January 2000–4 April 2020 (n = 25)

Study and publication year	Country	Study period	Study design	Selection criteria		Sex		Age at enrolment (years)	Number cases /controls, reports, or doses	Participants	Follow up/ time-window after vaccination		Person-years	Conflict of Interest / study sponsorship	Statement
				Inclusion	Exclusion	Female %	Male %								
Deceuninck et al. 2018 [36]	Canada	1999–2014	Retrospective ecological population-based	GBS as a main diagnosis, hospitalised	Only the first hospitalisation in a patient was retained	52	48	7–17	100 cases/ background rate	NA			13,736,161	Quebec Ministry of Health and Social Services	Author received grants from GSK and Pfizer and travel reimbursement to attend an ad hoc advisory board meeting of GSK
Chao et al. 2012 [37]	US	2006–2008	Cohort	All ages who received one dose or more of the 4-valent recombinant vaccine, with ≥ 12 month health plan membership before vaccination	< 12 month health plan membership before vaccination	100	NA	99% was 9–26	NA	561,457; 149,306 vaccinated/ 412,151 controls	180 days		648,821	Merck and Co	Funded and employed by pharmaceutical companies which had significant input into the study
Gee et al. 2011 [38]	US	2006–2009	Cohort (VSD)	Identified at the seven VSD sites	NR	100	NA	9–26	600,558 vaccine doses / background rate	NA	42 days		NR	Supported through ‘Vaccine Safety Surveillance and Assessment Projects contract’ with America’s Health Insurance Plans, funded by US CDC	The authors report research support by GSK, Merck and Co, Sanofi Pasteur, Wyeth (Pfizer), Novartis, and MedImmune now AstraZeneca
Gee et al. 2017 [39]	US	2006–2015	Cohort (VSD)	One dose or more of the 4-valent recombinant vaccine	NR	68.5 (calculated)	31.5 (calculated)	9–26	2,773,185 doses/ background rate	NA	42 days		NR	US CDC study	“*The findings and conclusions in this report are those of the authors and do not necessarily represent the official position of the CDC.*”
Donahue et al. 2019 [40]	US	2015–2017	Cohort (VSD)	One dose or more of the 4-valent recombinant vaccine	Doses < 42 days of a previous dose in the same person	47.6	52.4	9–29	128,645 vaccinated; 431,401 historical controls	560,046	Up to 180 days		NR	US CDC study	Author(s) report research support from Merck (including a HPV4 vaccine phase 4 post-marketing safety study), Sanofi Pasteur, GSK, Protein Science (now Sanofi Pasteur), and Pfizer
Slade et al. 2009 [41]	US	2006–2008	Cohort (VAERS)	Reported between June 2006 and December 2008	NR	97	3	6–29	12,424 reports / background rate	NA	0–145 days; 6 weeks (4–42 days) considered biologically plausible		NR	US CDC and FDA study	The authors declare no financial conflicts of interest; CDC was directly involved in all aspects of the study
Souayah et al. 2011 [42]	US	2006–2009	Case–control (VAERS)	Reported between June 2006 and September 2009	NA (case–control study)	NR licensed for women only		Mean: 16.7 (SD: 6.2)	31,819 reports; 13,115 HPV-vaccinated/ 13,801 other vaccination	NA	≤ 6 weeks		NR	NR	NR
Geier et al. 2015 [34]	US	2006–2012	Case–control (VAERS)	Listed US residence	NA (case–control study)	100	0	18–39	22,011 reports	NA	NR			Non-profit 501(c)(3) Institute of Chronic Illnesses Inc, by a grant from the Dwoskin Family Foundation	The authors declare they have no conflicts of interest
Ojha et al 2014 [43]	US	2010–2012	Cohort study (VAERS)	All reports on the 4-valent recombinant vaccine or other vaccinations among females and males aged 9–26 years	NR	63%	37%	9–26	14,822 reports; 4,670 HPV-vaccination reports / 10,152 other vaccine reports	NA	5–42 days	NR		Authors were supported by the American Lebanese Syrian Associated Charities, National Cancer Institute awards to the University of Alabama at Birmingham Comprehensive Cancer Center	The authors declare no financial or non-financial competing interests
Geier et al. 2017 [35]	US	2006–2014	Case–control (VAERS)	Listed US residence	NA (case–control study)	100	NA	6–39	48,852 reports	NA	NR			Non-profit 501(c)3 Institute of Chronic Illnesses	The authors declare that they have no conflicts of interest
Arana et al. 2018 [44]	US	2009–2015	Cohort (VAERS)	All reports on the 4-valent recombinant vaccine between 2009 and 2015	NA	60.2 22.6% sex unknown	17.2	11–17 (40.7%); unknown (42.2%)	19,760 4-valent recombinant vaccination reports/ 60,461,220 doses distributed	NA	Partially according to Brighton criteria		NA	US CDC study	None
Neha et al. 2020 [45]	US	2009–2015	Cohort (VAERS)	All reports on HPV vaccination between 2006 and 2017	NA	NR			49,444 reports	NA	NR		NA	None	None
Lehtinen et al. 2016 [24]	Finland	2007–2010	Community-randomised controlled trial	33 major, non-adjacent Finnish communities, Finnish or Swedish-speaking; 1992–1995 birth cohorts	NR	63.8 (calculated)	36.2 (calculated)	12–16 Mean: 14.1 (SD: 0.76)	NA	32,176	12 months		NR	GSK funded	GSK involved in all stages of the study and analysis
Bi et al. 2018 [25]	Finland	2007–2010	Community-based RCT	33 major, non-adjacent Finnish communities, Finnish or Swedish speaking; 1992–1995 birth cohorts	NR	63.8 (calculate)	36.2 (calculated	12–16 Mean: 14.1 (SD: 0.76)	NA	32,175	Up to 6.5 year		NR	GSK funded	GSK involved in all stages of the study and analysis
Skufca et al. 2018 [26]	Finland	2013–2016	Nationwide population-based observational retrospective register cohort	Females	Persons who have been vaccinated in clinical trials before the NVP were included in this study (birth cohorts 1992–1995)	100	NA	11–15	NA	240,605; 134,615 vaccinated/ 105,990 controls	0–180/181–365/ > 365 days		431,075	National Institute for Health and Welfare, GSK and Pfizer	Authors received grants from several pharmaceutical companies
Grönlund et al. 2016 [27]	Sweden	2006–2012	Register-based open cohort	All girls and women living in Sweden, diagnosed with one or more autoimmune diseases	Vaccinated before the start of individual follow-up, those who had died or emigrated before the start of follow-up	100	NA	10–30	NA	70,265	180 days after each dose		253,655	Swedish Foundation for Strategic Research and the Strategic Research Area in Epidemiology	Authors received grants from several pharmaceutical companies
Hviid et al. 2018 [28]	Sweden and Denmark	2006–2013	Register-based cohort	All women living in Sweden and Denmark	Bivalent vaccination	100	NA	18–44	NA	3,126,790; 8% vaccinated/ 92% controls	0–179/ ≥ 180 days/any		16,386,459	Novo Nordisk Foundation and Strategic research Areas, Karolinska Institutet, Danish Medical Research Council	Authors received grants from several pharmaceutical companies
Frisch et al. 2018 [29]	Denmark	2006–2016	National cohort	All boys born in Denmark in 1988–2006	GBS diagnosis before study entry	NA	100	Mean: 11.8 (SD: 2.5)	0	7,384	0–180/ > 180 days		4,200,000	Danish Medicines Agency; Danish Cancer Society	None
Grimaldi-Bensouda et al. 2014 [30]	France	2007–2011	Case–control	113 specialised centres recruited incident cases of six types of autoimmune disorders (incl. GBS)	NR	100	NA	14–26	Total: 1,365; 269 definite and probable cases / 1,096 controls GBS: 106; 15 cases/91 controls	NA	≤ 2 months		NR	Sanofi Pasteur MSD	NR
Grimaldi-Bensouda et al. 2017 [31]	France	2007–2014	Case- control	Living in France; able to undergo a telephone interview in French (participants or parents)	Patients with a lifetime history of autoimmune disease suspected at inclusion	100	NA	Median cases: 20.1 years/ referents 19.9 years	Total: 2,463; 478 cases/ 1,869 referents GBS: 143; 13 cases/130 referents	NA	≤ 42 days		NR	Financial involvement of GSK	Authors received grants from several pharmaceutical companies
Miranda et al. 2017 [13]	France	2008–2013	Longitudinal observational cohort	All girls aged 13–16 years covered by the general insurance scheme	History of HPV vaccination; any of the autoimmune diseases of interest before entry to the cohort.	100	NA	Median: 13.5 (SD: 0.87)	NA	2,252,716; 842,120 vaccinated/ 1,410,596 controls	Mean: 33 months		6,139,981	NR	None
Andrews et al. 2017 [32]	UK (England)	2007–2016	Self-controlled case series	Admissions with any mention of ICD-10 code for GBS (G610) in any of the 20 diagnosis fields	NR	100	NA	11–19	NA	100	Up to 365 days 0–91/92–183/184–365/0–1837 0–365		NR	Public Health England	None
Cameron et al. 2016 [23]	UK (Scotland)	2004–2014	Ecological	Hospital admissions in Scotland, a selection of 60 conditions	NR	NR; females and males male as controls		12–18	NR		NA; rate per population		NR	None	None
Willame et al. 2016 [33]	UK	2005–2010	Pooled analysis of observational cohort studies (CPRD GOLD)	One exposed female cohort and three unexposed cohorts: historical female, concurrent male, and historical male	De-enrolment date (death date or date of lost to follow-up) occurred before the study start date	Four cohorts: two female cohorts, two male cohorts		9–25 mean range: 15.3–16.0 (SD: 2.0–2.1)	NA	259,879; 64,705 vaccinated/ 194,192 controls	1–21 months		259,273	GSK Biologicals SA	GSK Biologicals SA designed the study, collected and analysed data, interpreted the results, and approved the manuscript
Verstraeten et al. 2008 [46]	NR	Up to mid-2007	Pooled analysis of RCT	All completed or ongoing RCT of AS04 adjuvanted HPV2, HSV and HBV vaccines conducted by GSK Biologicals or collaborators	New investigational HPV vaccine in an early development phase, for which only limited safety data were available	16 trials: 15 only females, one with males and females		10–72	NA	Total: 68,512 HPV-vaccinated: 39,160; 19,732 vaccinated/ 19,437 controls	Mean: 21.4 months		NR	GSK Biologicals	All authors are employed by GSK Biologicals; no other conflicts declared

CPRD GOLD: Clinical Practice Research Datalink General Practice Online Database; FDA: the United States Food and Drug Administration; GBS: Guillain–Barré syndrome; GSK: GlaxoSmithKline; incl: including; NA: not applicable; NR: not reported; RCT: randomised controlled trial; VAERS: Vaccine Adverse Event Reporting System; VDS: Vaccine Safety Datalink; SD: standard deviation; UK: United Kingdom; US: United States; US CDC: United States Centers for Disease Control and Prevention.

While most study populations comprised of adolescents, older and younger participants were also included. Age ranged from 6–72 years. We planned for inclusion of people aged 9 years and older [14] because the HPV vaccines are approved for people aged 9 years and older. However, for completeness, we decided not to exclude studies reporting on a broader age group. The majority of studies reported exclusively on the vaccination of girls and women. Twelve studies reported exclusively on females (100% females in 11 studies and 97% females in one study), nine studies reported on both sexes (some of which had males only as comparator), one study reported exclusively on males and three studies did not report the sex of the participants (Table 1).

The included studies comprised data of more than 10 million reports in total. Of 25 studies, 14 reported the number of cases/controls, reports or vaccine doses (range: 4,133,370–4,415,894), 10 studies reported the number of participants (range: 6,622,607–6,843,326) and one study [23] did not report any number of participants, reports or case/control numbers.

Three studies had a randomised design: two studies reported on the same community-based randomised controlled trial [24,25] and one was a pooled analysis of RCT [46]. The remaining 22 studies had a non-randomised design [13,23,26-45]: 14 cohort studies, five case–control studies, two ecological studies and one self-controlled case series. The cohort studies were based on either registry data, adverse event notification data and/or clinical or hospital databases.

#### Intervention

Fourteen of 25 studies reported exclusively on vaccination with the 4-valent recombinant vaccine [27-30,34-39,41-44] and five reported exclusively on the bivalent vaccine [24-26,33,46] (Table 2). Three studies reported on both the 4-valent recombinant vaccine and the bivalent vaccine [13,31,32], while one reported only on the 9-valent vaccine [40]. Two studies did not specify the type of HPV vaccine used [23,45].

**Table 2:** Occurrence of Guillain–Barré syndrome and association between Guillain–Barré syndrome and vaccination status, 1 January 2000–4 April 2020 (n = 25)

Study and publication year	Study design	Vaccine	Comparator	Outcome	Brighton criteria	After HPV vaccination			Comparator			Ratio/comparison	
				GBS diagnosis		Person-years	Number of cases of GBS	Incidence rate	Person-years	Number of cases of GBS	Incidence rate	Estimator	Confounders corrected for
Deceuninck et al. 2018 [36]	Retrospective ecological population-based	4-valent recombinant	No vaccine; non-targeted boys and girls	ICD-9 (357.0) or ICD-10 (G61.0) as main diagnosis	No	Age 7–8: NA	NA	NA	Girls: 1,190,724; Boys: 1,247,827	Girls: 6 Boys: 3	Girls: 0.50 Boys: 0.24/100,000 py	Adjusted RR: 0.81 (95% CI: 0.29–2.26)	Sex, age, year of GBS diagnosis, H1N1 influenza pandemic period
						Age 9: 211,291	1	0.47 (0.01–2.64)/ 100,000 py	Girls: 402,129 Boys: 642,494	Girls: 2 Boys: 1	Girls: 0.50 Boys: 0.16/100,000 py		
						Age 10–13: NA	NA	NA	Girls: 2,539,304 Boys: 2,658,862	Girls: 15 Boys: 19	Girls: 0.59 Boys: 0.71/100,000 py		
						Age 14: 222,751	1	0.45 (0.01–2.50)/ 100,000 py	Girls: 433,953 Boys: 685,925	Girls: 2 Boys: 5	Girls: 0.46 Boys: 0.73/100,000 py		
						Age 15–17: 124,953	2	1.60 (0.19–5.78)/ 100,000 py	Girls: 1,590,104; Boys: 17,85,852	Girls: 23 Boys: 20	Girls: 1.45 Boys: 1.12/100,000 py		
Chao et al. 2012 [37]	Cohort	4-valent recombinant	No vaccine	ICD-9 (original and expanded)	No	NR	0	NA	NR	0	NR	ND	NA
Gee et al. 2011 [38]	Cohort (VSD)	4-valent recombinant	Historical background rate using HCUP data	ICD-9 (357.0)	Yes	NR	0	NA	NR	0	Historical background IR (per 100,000 py), per age group (years): 9–10: 0.945; 11–14: 1.257; 15–17: 2.130; 18–26: 2.251	NR	NA
Gee et al. 2017 [39]	Cohort (VSD)	4-valent recombinant	Published background rate	ICD-9 (357.0)	Yes	NR	One case/2,773,185 vaccinations	Cumulative incidence for 1–42 day interval: 0.36	NA	Expected number of cases per one million vaccine doses	0.55 chart-confirmed GBS cases/ 100,000 py among a population aged 11–18 years	Not reported; attributable risk: 0	NA
Donahue et al. 2019 [40]	Cohort (VSD)	9-valent	Historical comparison (2007–2014), concurrent comparison	ICD-10 (G61.0)	No	NR	0	0	NR	3	NA	Not possible	NA
Slade et al. 2009 [41]	Cohort (VAERS)	4-valent recombinant	Background rate for females aged 9–26 years (based on Healthcare Cost and Utilisation Project data for 2000–2004)	MedDRA term GBS or text containing GBS or Guillain- Barré	Yes	NR	12 cases by Brighton criteria	Reporting rate 0.3/ 100,000 py	NR	Background rate of GBS among 9–26-year-old females	Reporting rate 1.57/100,000 py	Proportional reporting ratio: 0.4 in 6–29-year-olds	ND
Souayah et al. 2011 [42]	Case–control (VAERS)	4-valent recombinant	Meningococcal vaccine/ influenza vaccine /general population (literature)	Keyword and subject term ‘Guillain–Barré syndrome’; clinical findings and diagnostic test results were reviewed by a board-certified neuromuscular specialist	No	NR	34 cases within the first 6 weeks	Weekly reporting rate in the first 6 weeks: 6.6/ 10,000,000	ND	Within the first 6 weeks: Meningococcal vaccine: 42; Influenza vaccines: 132; average annual incidence general population: 34–134/10,000,000	Weekly reporting rate in the first 6 weeks: Meningococcal vaccine: 3/10,000,000; Influenza vaccine: 1.3/10,000,000; average weekly incidence general population: 0.65–2.57/10,000,000	ND *“Nearly a 2.5 to 10-times greater risk of acquiring GBS within 6 weeks after Gardasil vaccination when compared to the general population. Compared to Menactra, the VAERS database reported more post-Gardasil GBS within the first 2 weeks post-Gardasil GBS within the first two weeks post-vaccination”*	NA
Geier et al. 2015 [34]	Cohort (VAERS)	4-valent recombinant	Controls: other (non-GBS) reported adverse events associated with the 4-valent recombinant vaccine	VAERS code: 10018767	No	NR	18 cases/ 5,106 controls	NR		79 cases/ 16,808 controls	NR	Unadjusted OR: 0.75 (95% CI: 0.42–1.3)	ND
Ojha et al. 2014 [43]	Cohort study: VAERS	4-valent recombinant	All other vaccinations	MedDRA term: Guillain– Barré syndrome	No	NR	Nine cases /4,670 reports (0.19%)	NR	36 cases /10,152 reports (0.35%)	NR		PRR: 0.54 (95% CI: 0.26–1.1)	ND
Geier et al. 2017 [35]	Cohort (VAERS)	4-valent recombinant	Controls: other (non-GBS) reported adverse events associated with the 4-valent recombinant vaccine	VAERS code: 10018767	No	NR	54 cases/15,330 controls	NA		140 cases/33,328 controls	NR	Unadjusted OR: 0.839 (95% CI: 0.601–1.45)	ND
Arana et al. 2018 [44]	Cohort (VAERS)	4-valent recombinant	Other reports	MedDRA terms: Guillain– Barré syndrome and clinical review	Yes	NR	14 by Brighton criteria	NA			NR	NA	NA
Neha et al. 2020 [45]	Cohort (VAERS)	NR	Other reports	MedDRA term Guillain–Barré syndrome	No	NA	26	NR			ND	Not possible	NA
Lehtinen et al. 2016 [24]	Community-based RCT	Bivalent	HBV-vaccination	ICD-10	No	NR	0	No case during 12 month follow up	NR	0	No case during 12 month follow up	Not possible	NA
Bi et al. 2019 [25]	Community-based RCT	Bivalent	HBV-vaccination	ICD-10	No	63,9327 years	0	Not possible	75,460.8 years	1	Not possible	Not possible	NA
Skufca et al. 2018 [26]	Nation-wide population-based observational retrospective register cohort study	Bivalent	Not vaccinated	ICD-10 (G610)	No	Overall: 186,934	Overall: 6	NR	244,141	1	NR	Overall: crude HR: 4.95 (95% CI: 0.58–41.88); adjusted HR: 5.31 (95% CI: 0.62–45.39)	Hospital district, country background (born abroad or in Finland) and number of any hospital visits or admissions two years before the scheduled vaccination
						0–180 days: 55,770	0–180 days: 2					0–180 days: crude HR: 2.64 (95% CI: 0.23–30.18); adjusted HR: 2.76 (95% CI: 0.24–32.04)	
						181–365 days: 48,332	181–365 days: 2					181–365 days: crude HR: 7.54 (95% CI: 0.55–102.9); adjusted HR: 8.27 (95% CI: 0.60–1113.7)	
						> 365 days: 82,832	> 365 days: 2					> 365 days: crude HR: 27.56 (95% CI: 1.41–538–8); adjusted HR: 32.17 (95% CI: 1.59–652.4)	
Grönlund et al. 2016 [27]	Register-based open cohort	4-valent recombinant	Unvaccinated	ICD-10 (G61.0)	No	7,848	0	0/7,845 py	245,807	6	IR: 0.02 (0.01 to 0.05)/1,000 py	Not possible	Not possible
Hviid et al. 2018 [28]	Register-based cohort	4-valent recombinant	No 4-valent recombinant vaccination	ICD-10 (G61.0)	No	319,298	0	IR: 0/100,000 py	16,067,162	194	IR: < 2/100,000 py	Not possible	Not possible
Frisch et al. 2018 [29]	National cohort	4-valent recombinant	No 4-valent recombinant vaccination	ICD-10 (G61.0)	No		0	NR				Not possible	ND
Grimaldi-Bensouda et al. 2014 [30]	Case–control	4-valent recombinant	No vaccination	‘Internationally accepted classification’ with reference to Brighton criteria	Yes	NR	0	NR		7	NR	Not possible	Not possible
Grimaldi-Bensouda et al. 2017 [31]	Case–control	Bivalent, 4-valent recombinant	Referents: no lifetime history of any of the ADs under study	‘Internationally accepted classification’ without reference to Brighton criteria	No	NR	0 cases/13 referents	NA	NR	Two cases/ 10 referents	NA	Not possible	NA
Miranda et al. 2017 [13]	Longitudinal observational cohort	Bivalent, 4-valent recombinant	Unvaccinated	ICD-10 (G61.0)	No	1,393228	20	Crude IR: 1.36/100,000 py	4,746,753 py	23	Age-standardised IR: 0.37	Unadjusted HR: 3.62 (95% CI: 1.73–7.59); adjusted HR: 3.78 (95% CI: 1.79–7.98)	Age (time scale), year of inclusion, geographical zone, CMU, history of use of healthcare and other vaccinations, use of healthcare and other vaccinations after inclusion
Andrews et al. 2017 [32]	Self-controlled case series	Bivalent and 4-valent recombinant	Self-controlled: same person, incidence in different risk timeframe	ICD-10 (G610)	No	NR	0–91 days; nine cases	RI: 1.04 (95% CI: 0.47–2.28)	NR	92–183 days: five cases	RI: 0.78 (95% CI: 0.27–2.21)	RI risk period 0–91 days: 1.04 (95% CI: 0.47–2.28)	Age, period and season
										184–365 days: 10 cases	RI: 1.41 (95% CI: 0.61–3.22)		
										0–183 days: 14 cases	RI: 0.83 (95% CI: 0.41–1.69)		
Cameron et al. 2016 [23]	Ecological study	NR	Population based: boys (not eligible for HPV vaccination in Scotland)	ICD-10 (G610, G611, G618, G619)	No	NR						Observed/ expected	NA
Willame et al. 2016 [33]	Pooled analysis of observational cohorts: CPRD GOLD	Bivalent	Unexposed cohorts	Medcode (CPRD-GOLD Medical Code Events): 1607 Read Code Read Description: F370000 ICD-10 codes: G61.0	No	64,705	0	IR: 0.00/100,000 py	Unexposed historical female cohort: 64,841 py	0	IR: 0.00/100,000 py	NP	NA
									Unexposed concurrent male cohort: 64,859 py	1	IR: 1.54/100,000 py		
									Unexposed historical male cohort: 64,868 py	1	IR: 1.54/100,000 py		
Verstraeten et al. 2008 [46]	Pooled analysis of RCT	Bivalent	Non-adjuvanted control vaccine, aluminium adjuvanted vaccines, or aluminium hydroxide alone	MedDRA	No	NR						ND; RR not calculated unless an event occurred in one group	NA

CI: confidence interval; CPRD GOLD: Clinical Practice Research Datalink General Practice Online Database; GBS: Guillain–Barré syndrome; GSK: GlaxoSmithKline; HR: hazard ration; ICD: International Classification of Diseases; IR: incidence rate; MedDRA: Medical Dictionary for Regulatory Activities; NA: not applicable; ND: not done; NP: not possible; NR: not reported; OR: odds ratio; py: person-year; RCT: randomised controlled trial; RI: relative incidence; RR: relative risk; VAERS: Vaccine Adverse Event Reporting System; VDS: Vaccine Safety Datalink.

#### Comparator

Studies compared the risk of occurrence of GBS after HPV vaccination to either the risk of GBS after no vaccine (e.g. non-targeted populations such as boys), to another vaccine e.g. hepatitis B (HBV vaccine), meningococcus or influenza vaccine), or against historical background rates (Table 2). Case–control studies compared frequency of HPV vaccination in GBS cases to the frequency of HPV vaccination in controls; the controls being the general population or other adverse events reported to the vaccine safety registry.

#### Outcome

The duration of follow-up time varied, ranging from 42 to 180 days post-vaccination to the total accumulated number of person-years available in the registry or medical records. Eight studies reported the total follow-up time [13,26-29,33,36,37], which adds up to 42,055,425 reported person-years in total.

While five studies referred to the Brighton criteria for GBS case definitions [30,38,39,41,44], in the majority of studies GBS diagnosis was based on original and expanded International Classification of Diseases codes (ICD-9 and ICD-10) as well as other coding systems such as Medcode, MedDRA and VAERS and free-text notes in medical files (Table 2).

#### Risk of bias

**Figure 2:**
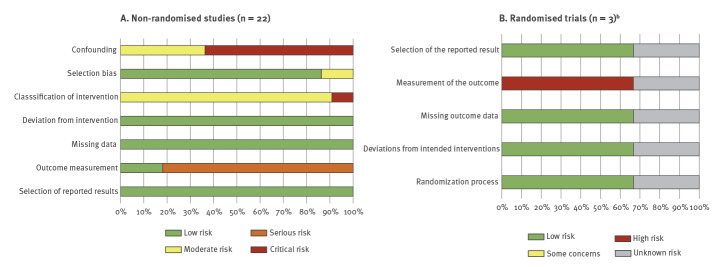
Summary of risk of bias assessment, 1 January 2000–4 April 2020 (n = 25)^a^

^a^ Full results are provided in Supplementary Table S4.

^b^ Two of three publications are based on the same community-randomised controlled trial (interim and end-of-study results) [24,25].

We summarised the findings of our risk of bias assessment in Figure 2 and Supplementary Table S3. The risk of bias in the community-based RCT by Lehtinen et al. and Bi et al. [24,25] was considered to be low for most indicators, but high regarding the selection of the reported results, which was limited for GBS. We were not able to assess the risk of bias in the pooled analysis of 42 studies by Verstraeten et al. [46], of which 16 reported HPV vaccination, because of the complexity of the pooled design and lack of reporting of the key indicators for the risk of bias assessment. The risk of bias in 14 of 22 non-randomised studies was assessed as being critical and eight studies were assessed as being at serious risk of bias. The risk of bias was mostly introduced by the critical risk of confounding (i.e. lack of confounding correction), or because of the outcome measurement i.e. GBS diagnosis not based on Brighton criteria [15]. Risk of bias because of the classification of the intervention (vaccination status) was moderate when based on a registry and critical when self-reported. Less than 10 studies per outcome and per study design were available for pooling, which prevented us from systematically assessing publication bias by means of a funnel plot.

### Results of individual studies

The reported occurrence of GBS following HPV vaccination is summarised in Table 2 and described by study design and by geographic region.

#### Randomised studies

Two randomised studies did not observe a single GBS case among people who received the HPV vaccine and found no increased risk of GBS after HPV vaccination. Lehtinen et al. [24] and Bi et al. [25] reported on a large community-based RCT in Finland. Among 32,176 adolescents, no GBS cases were diagnosed after the bivalent vaccine (or HBV vaccination). Verstraeten et al. [46] described the findings of a pooled analysis of all RCT of AS04-adjuvanted bivalent vaccines, HSV and HBV vaccines. One GBS case was observed among 68,512 participants, in the control group.

#### Non-randomised studies

One cohort study [13] and two case–control studies [30,31] investigated the potential association between the HPV vaccine and autoimmune disease in France. In the cohort study of more than two million girls by Miranda et al. [13], an increased risk of GBS was observed among vaccinated girls. There was an IR of 1.36 cases per 100,000 person-years among vaccinated individuals (20 cases), compared with 0.37 cases per 100,000 person-years among unvaccinated individuals (23 cases), with an adjusted hazard ratio (aHR) of 3.78 (95% CI: 1.79–7.98). The association was particularly marked in the first 2 months after vaccination and decreased over time, and did not differ with the type of HPV vaccine or whether or not GBS was preceded by a recent history of gastrointestinal or respiratory tract infection. Seasonality and calendar year did not affect the findings. In the two included case–control studies, no exposure to HPV vaccine was observed in cases with GBS [30,31].

A cohort study with 3,126,790 Swedish and Danish women [28] did not observe a single case of GBS among those who received the 4-valent recombinant vaccine (319,298 person-years); 194 cases were observed among the unvaccinated (16,067,162 person-years). Grönlund et al. [27] studied a cohort of 70,265 girls and women with pre-existing autoimmune disease in Sweden. None of those who received the 4-valent recombinant vaccine developed new-onset GBS (7,845 person-years); six cases of GBS were observed among the unvaccinated (245,807 person-years); the IRR could not be calculated. Frisch et al. [29] studied 7,384 Danish boys born in 1988–2006. No case of GBS was observed after the 4-valent recombinant vaccine during the four million person-years of follow up in 2006–2016.

In Finland, Skufca et al. [26] investigated baseline annual, pre-vaccination, and post-vaccination period incidences of GBS, followed by a nationwide population-based observational cohort study among 240,605 women that compared the risk of GBS between those exposed and not exposed to the bivalent vaccine. There was no increase in GBS incidence in the post-vaccination period in neither men nor women. Cox regression showed a statistically non-significant trend towards increased risk of GBS after HPV vaccination among women, with an aHR of 5.31 (95% confidence interval (CI): 0.62–45.39). A year after vaccination, the aHR was substantially increased at 32.17 with a wide CI (95% CI: 1.59–652.4).

A self-controlled case series from England [32] compared GBS incidence following vaccination with the bivalent and 4-valent recombinant vaccines in different risk timeframes. The relative incidence was 1.04 (95% CI: 0.47–2.28) for the 3-month risk period and 0.83 (95% CI: 0.41–1.69) and 1.10 (95% CI: 0.57–2.14) for the 6- and 12-month period, respectively. Based on this finding the authors excluded a risk in the order of one GBS case per million HPV doses.

Both ecological studies, in Scotland [23] (trend analysis, no data extraction possible) and in Canada [36] (aRR: 0.81; 95% CI: 0.29–2.26), observed no increased risk of GBS following HPV vaccination.

Eleven studies reported data from the US: seven studies based on the Vaccine Adverse Event Reporting (VAERS) [47] registry, three based on the Vaccine Safety Datalink (VSD) safety monitoring data [48] and one cohort study. Seven studies reported 2006–2017 data on HPV vaccination from the VAERS registry.

A descriptive summary of 12,424 VAERS reports between 2006 and 2008 by Slade et al. [41] reported 0.3 confirmed GBS cases per 100,000 person-years following the 4-valent recombinant vaccine, compared with 1.57 cases per 100,000 person-years following all other vaccinations in the same age group. The proportional reporting ratio for GBS after the 4-valent recombinant vaccine was 0.4 and did not meet the screening criteria for signal detection. Arana et al. [44] provided an update of the analysis, and summarised 19,760 VAERS reports from 2009 to 2015. In total, 59 reports of GBS have been identified, of which 14 (24%) met the Brighton Collaboration criteria at Level 1 (n = 5) which is the highest level of diagnostic certainty, or Level 2 (n = 9). The 4-valent recombinant vaccine was given alone in six reports. The crude reporting rate of GBS was 0.98 per one million 4-valent recombinant vaccine doses distributed, based on an estimated 60,461,220 doses distributed in the US in that time period (2009–2015). Additionally, Neha et al. [45] reviewed all clinically relevant vaccine event combinations following HPV vaccination as reported to VAERS between 2006 and 2017; no safety concern was identified. Souayah et al. [42] compared the weekly reporting rates of GBS, in the 6 weeks after vaccination against HPV genotype 4 (6.6 cases per week/10,000,000 people), meningococcal vaccine (3 cases per week/10,000,000 population), and influenza vaccine (1.3 cases per week/10,000,000), based on VAERS reports between 2006 and 2009. Furthermore, these rates were compared with the expected weekly incidence in the general population (0.65–2.57 cases per week/10,000,000) based on a literature review. The authors summarise these findings as *“There was nearly a 2.5-to 10-times greater risk of acquiring GBS within 6 weeks after Gardasil vaccination when compared with the general population.”* [42]. For the period of 2010–2012, Ojha et al. [43] compared the reporting of GBS cases to VAERS following vaccination with the 4-valent recombinant vaccine (nine cases/4,670 reports; 0.19%) to those of other vaccines (36 cases/10,152 reports; 0.35%) with a proportional reporting ratio of 0.54 (95% CI: 0.26–1.1).

Two case–control studies were based on data from 22,011 VAERS reports in the period of 2006–2012 targeting 18–39-year-old women [34] and on 48,852 reports in 2006–2014 targeting 6–39-year-old girls and women [35]. Both studies found no association between GBS and the 4-valent recombinant vaccine, with an unadjusted OR of 0.75 (95% CI: 0.42–1.3) and 0.84 (95% CI: 0.60–1.15), respectively.

Three studies reported on 2006–2017 VSD safety monitoring data of the bivalent vaccine and the 4-valent recombinant vaccine [38-40]. Two studies on the 4-valent recombinant vaccine based on the 2006–2015 VSD safety monitoring data found lower GBS incidences following vaccination, compared with the background rates. Gee 2011 et al. [38] observed one case of GBS among the 600,558 4-valent recombinant vaccine doses administered between 2006 and 2009, of which medical record review revealed that this was not an incident case. Gee 2017 et al. [39] observed one case of GBS among 2,773,185 4-valent recombinant vaccine doses administered between 2006 and 2015 (IR: 0.36/1,000,000 doses; one-sided 95% CI: 1.71). Donahue et al. [40] reported on safety data of the 9-valent vaccine based on 128,645 doses given between 2015 and 2017, and did not observe GBS among the vaccinated; three cases of GBS were reported in the historical comparison group.

Additionally, Chao et al. [37] conducted a cohort study in California among 189,629 women who received one dose or more of the 4-valent recombinant vaccine in the period of 2006–2008 and did not observe a single GBS case among both vaccinated and unvaccinated women.

### Meta-analysis

Seven studies reported an effect-estimate suitable for meta-analysis [13,26,32,34-36,43]. The remaining 18 studies were not suitable because only descriptive outcomes were available, or because one or both groups did not observe a single case. Two studies by Geier et al. published findings with overlap in data based on the VAERS dataset [34,35], with similar findings. The effect estimate by Geier et al. 2017 [35] was used in the primary analyses and the Geier et al. 2015 [34] estimate in the sensitivity analysis. Therefore, the results of the meta-analysis show findings of six studies.

**Figure 3:**
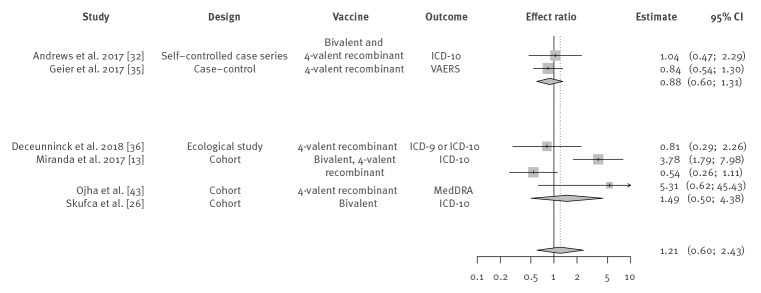
Meta-analysis of studies reporting an effect estimate of the risk of Guillain–Barré syndrome after HPV vaccination, by study design, 1 January 2000–4 April 2020 (n = 7)^a,b^

CI: confidence interval; ICD: International Statistical Classification of Diseases and Related Health Problems (International Classification of Diseases); VAERS: vaccine adverse event reporting system.

^a^ Meta-analysis yielded a pooled random-effects model ratio of HPV vaccination on GBS of 1.21 (95% CI: 0.60–2.43); I^2^ = 72% (95% CI: 36–88).

^b^ Two studies published findings with a large overlap in data based on the VAERS dataset [34,35] with similar findings. The effect estimate by Geier et al. 2017 [35] was used in the primary analyses and Geier et al. 2015 [34] estimate in the sensitivity analysis. Therefore, the results of the meta-analysis show findings of six studies. When substituting the effect estimate of Geier et al. 2017 for Geier et al. 2015 (OR: 0.75; 95% CI: 0.42–1.3), the pooled estimate for case–control studies is 1.19 (95% CI: 0.58–2.43), I^2^ = 72% (95% CI: 37–88).

Meta-analysis yielded a pooled random-effects model ratio of HPV vaccination on GBS of 1.21 (95% CI: 0.60–2.43); I^2^ = 72% (95% CI: 36–88) (Figure 3). Sensitivity analysis including Geier et al. 2015 [34] instead of Geier et al. 2017 [35], yielded similar results: 1.19 (95% CI: 0.58–2.43); I^2^ = 73% (95% CI: 37–88). The pooled estimate was 0.88 (95% CI: 0.60–1.31) for self-/case-controlled studies and 1.49 (95% CI: 0.50–4.38) for cohort studies. Findings of the subgroup analysis by vaccine type (bivalent vaccine, 4-valent recombinant vaccine or both) and outcome measure are provided in the Supplement.

All effect (adjusted) effect estimates (odds ratios, hazard ratios, relative risks, proportional reporting ratio, relative incidence, incidence rate ratio) are combined in one overall ratio.

Additional subgroup analysis by Brighton vs non-Brighton GBS case definition was not possible because all seven studies used non-Brighton criteria. The included studies were heterogeneous in terms of study design, analysis and reporting, which was also reflected in the high I^2^, which indicates the percentage of variability in the effect sizes which is not caused by sampling error.

### Quality of the body of evidence

According to the GRADE approach, the quality of the body of evidence is very low. We had to downgrade the quality of evidence three times: (i) for risk of bias (residual confounding at least); (ii) for imprecision (wide 95% CI around the pooled estimate including substantial benefit as well as substantial harm) and; (iii) heterogeneity (inconsistency; I^2^ = 72%).

### Number needed to harm and number needed to vaccinate

Translating the pooled random-effect ratio of 1.21 (95% CI: 0.60–2.43) to the number needed to harm (NNH), we estimated that one million people need to take the HPV vaccine to generate one case of GBS (95% CI: − 3 to 8 cases). In contrast, 324 (80% credibility interval: 195–757) people need to be vaccinated to prevent one cases of cervical cancer [48].

## Discussion

The results of our systematic review and meta-analysis indicate that absolute risk of GBS after HPV vaccination is low. Reported historical background rates of GBS incidence were between 0.55 and 2.25 cases per 100,000 person-years [38,39]. A slightly increased RR of GBS after HPV vaccination is low, far away from statistical significance based on findings from our meta-analysis. From a public health point of view, up to one million people would need to be vaccinated to generate one additional case of GBS, while the NNV to prevent one case of cervical cancer is ca 300.

We performed a comprehensive and systematic search on this topic that includes all licenced HPV vaccines. It was specifically targeted at investigating the association with GBS, in the context of other reviews that focussed on the potential association between HPV vaccination and autoimmune and/or neurological diseases [49-51].

The quality of our findings depended on the quality of the studies, which were largely registry studies and based on non-Brighton GBS outcomes. There was risk of bias because of large heterogeneity in the design and reporting of the studies, as well as the control groups. Confounding was the biggest limiting factor of the quality of the evidence, because many studies were not designed to correct for confounding and cohorts were highly confounded. Often, the control group was not matched based on sex or age group. Outcome ascertainment was challenging, given the heterogeneity of case definitions for GBS; only one of five studies used the Brighton criteria.

The follow-up period for the detection of GBS varied between studies and many of the cohorts or registry-based studies were partially underpowered by design because of the rarity of GBS. In the three studies that signalled increased risk of GBS after HPV vaccination, conflicting temporal trends were reported. In the study by Miranda et al. [13] the association between vaccination and GBS was particularly marked in the first 2 months after vaccination and decreased over time, while Skufca et al. [26] reported a substantial increase in the association (with very wide CI). Souayah et al. [29] also noted increased reporting of GBS during the first 6 weeks after vaccination, although interpreting this trend was challenging in this VAERS reporting study since all five other studies reporting VAERS data found no association between HPV vaccination and GBS. Interpretation of these results in terms of causality should be made with caution.

In future studies, consensus on the case definition for GBS and the risk timeframe is needed to generate uniform and comparable findings. Studies in settings with gender-neutral vaccination policies are required to further assess the risk of GBS after HPV vaccination among boys and men. Furthermore, studies should be expanded to geographical areas outside of western Europe and North America, where HPV vaccination is being implemented on large scale. To generate the highest quality of evidence on this topic, we recommend further research with a self-controlled case series design using Brighton-outcomes. The self-controlled case series design has been proved most suitable for rare events and limits confounding to time-dependent confounding [21], as also used to assess the risk of intussusception after rotavirus vaccination [52].

This study aims to provide up to date vaccination safety information for healthcare providers and policymakers as well as the general public [53]. Transparent communication of potential safety issues is essential to build trust and strengthen confidence in HPV vaccination. Concern about vaccine safety is one of the key determinants of vaccine hesitancy and poses a threat to public health. Healthcare providers play an important role in communicating information on HPV safety [54]. The low potential risk of GBS after HPV vaccination should have minimal impact on the risk consideration for HPV vaccination programmes, reassure vaccine confidence and ultimately increase vaccination rates.

## Supplementary Data

313000 Supplement
