# Supplementary material for: Risk of Guillain–Barré syndrome after vaccination against human papillomavirus: a systematic review and meta-analysis, 1 January 2000 to 4 April 2020
Source: Euro Surveill. 2022 Jan 27;27(4):2001619. doi: 10.2807/1560-7917.ES.2022.27.4.2001619 (PMC8796292; doi:10.2807/1560-7917.ES.2022.27.4.2001619)
Supplement: Supplement [file 20-01619_BOENDER_Supplement.pdf]

Supplementary Material

This supplementary material is hosted by Eurosurveillance *as supporting information alongside the article "Risk of Guillain–Barré syndrome after vaccination against human papillomavirus: a systematic review and meta-analysis, 1 January 2000 to 4 April 2020", on behalf of the authors, who remain responsible for the accuracy and appropriateness of the content. The same standards for ethics, copyright, attributions and permissions as for the article apply. Supplements are not edited by* Eurosurveillance *and the journal is not responsible for the maintenance of any links or email addresses provided therein.*

Supplement 1: Search strategy

A) Embase Session

Results on 21 January 2019

No.	Query	N Results
#24	#23 AND 'human'/de AND (2000:py OR 2001:py OR 2002:py OR 2003:py OR 2004:py OR 2005:py OR 2006:py OR 2007:py OR 2008:py OR 2009:py OR 2010:py OR 2011:py OR 2012:py OR 2013:py OR 2014:py OR 2015:py OR 2016:py OR 2017:py OR 2018:py OR 2019:py)	487
#23	#5 AND #15 AND #22	516
#22	#16 OR #17 OR #18 OR #19 OR #20 OR #21	595,833
#21	fisher AND syndrom*	9,639
#20	gbs	10,278
#19	'guillain' AND 'barr*' AND syndrom*	17,201
#18	'guillain-barré' AND syndrom*	17,126
#17	'autoimmune disease'/exp	579,374
#16	'guillain barre syndrome'/exp	13,866
#15	#6 OR #7 OR #8 OR #9 OR #10 OR #11 OR #12 OR #13 OR #14	549,520
#14	cervarix	1,748
#13	silgard	102
#12	gardasil	2,384
#11	'wart virus vaccine'/exp	12,052
#10	'virus vaccine'/exp	147,639
#9	vaccin*	481,097
#8	'vaccine'/exp	321,686
#7	'immunization'/exp	276,188
#6	'vaccination'/exp	160,716
#5	#1 OR #2 OR #3 OR #4	79,489
#4	human AND papillomavirus	48,061
#3	hpv	50,977
#2	'wart virus'/exp	33,384
#1	'papillomavirus infection'/exp	29,310

Results on 2 April 2020

No.	Query	N Results
#24	#23 AND 'human'/de AND (2018:py OR 2019:py OR 2020:py)	93
#23	#5 AND #15 AND #22	565
#22	#16 OR #17 OR #18 OR #19 OR #20 OR #21	640,191
#21	fisher AND syndrom*	10,872
#20	gbs	11,507
#19	'guillain' AND 'barr*' AND syndrom*	18,328
#18	'guillain-barré' AND syndrom*	18,249
#17	'autoimmune disease'/exp	621,664
#16	'guillain barre syndrome'/exp	14,751
#15	#6 OR #7 OR #8 OR #9 OR #10 OR #11 OR #12 OR #13 OR #14	587,771
#14	cervarix	1,834
#13	silgard	111
#12	gardasil	2,520
#11	'wart virus vaccine'/exp	13,575
#10	'virus vaccine'/exp	157,233
#9	vaccin*	516,205
#8	'vaccine'/exp	342,707
#7	'immunization'/exp	295,297
#6	'vaccination'/exp	174,046
#5	#1 OR #2 OR #3 OR #4	87,139
#4	human AND papillomavirus	53,263
#3	hpv	56,651
#2	'wart virus'/exp	35,907
#1	'papillomavirus infection'/exp	34,527

B) Search strategy - PubMed Results on 2 April 2020

**(**"Alphapapillomavirus"[Mesh]<br>OR<br>hpv<br>OR (human AND papillomavirus)**)**
**AND**
**(**"Papillomavirus Vaccines"[Mesh]<br>OR<br>"Vaccines"[Mesh]<br>OR<br>"Vaccination"[Mesh]<br>OR<br>"Human Papillomavirus Recombinant Vaccine Quadrivalent, Types 6, 11, 16, 18"[Mesh]<br>OR<br>"Immunization"[Mesh]<br>OR<br>(cervarix OR silgard OR gardasil)<br>OR<br>vaccine<br>OR<br>vaccination**)**
**AND**
**(**"Guillain-Barre Syndrome"[Mesh]<br>OR<br>"Miller Fisher Syndrome"[Mesh]<br>OR<br>"Autoimmune Diseases"[Mesh]<br>OR<br>"Autoimmune Diseases of the Nervous System"[Mesh]<br>OR<br>(fisher AND syndrom*)<br>OR<br>gbs<br>OR<br>(guillain AND (barré or barre) AND syndrom*)<br>OR<br>(guillain-barré AND syndrom*)**)**

Comments: Search is largely similar to Embase search. Replaced ‘vaccin’ and 'barr*' with (‘vaccine’ or ‘Vaccination’) and (barré or barre), to avoid a wildcard search.*

Full search: 152 hits.

Publication date from 2018/01/01: 22 hits.

Supplement 2: Data extraction sheet

**Study meta-data**	SHORT	DETAIL
**Study** (firstauthor_year)		
**Reference**		
**Title**		
**Authors**		
**Year of publication**		
**Journal of publication**		
**DOI**		
**Study design**	SHORT	DETAIL
**Location of the study** (country)		
**Study period** (calendar years)		
**Study design**		
**Inclusion criteria**		
**Exclusion criteria**		
**- If case-control:**<br>**> How are the controls selected?**<br>**> How are the controls matched?**<br>**>> Case-control ratio**<br>**>> Characteristics used for matching**		
**Population**	SHORT	DETAIL
**Sex** (% women)		
**Age at enrollment** (in years)		
**Number of people included** (total)		
**- Final no. of participants** (analyzed)		
**- N vaccinated**		
**- N controls**		
**Length of folow-up**		
**- Follow up time vaccinated**		
**- Follow up time controls**		
**- If cohort study:**<br>**> How many (%) were loss to follow-up?**		
**Intervention & Control**	SHORT	DETAIL
**Vaccine name** (Gardasil or Cervarix)		
**- Number of vaccine doses**		
**Vaccination register or reimbursement**<br>(self-reported, organisation)		
**Definition of control group**<br>(e.g. placebo, no vaccination, other vaccination)		
**Co-interventions**<br>(optional; potential confounding factor)		
**Outcome**	SHORT	DETAIL
**GBS outcome Case definition used for Guillain-Barré Syndrome (GBS)**		
**- Brighton or non-Brighton GBS case definition**		
**- Source of outcome reporting**<br>(e.g. ICD-9/10; register data, self-reported, hospital data, GP database)		
**VACCINATED. Incidence of GBS reported**		

**CONTROLS. Incidence of GBS reported**		
**Unadjusted hazard ratio/odds ratio/relative risk (uHR, uOR, uRR) with 95% conficende interval (95%CI)**		
**Adjusted odds ratio/relative risk (aOR, aRR) with 95% confidence interval (95%CI)**		
**- Confounders considered in adjustment**		
**Risk of bias**	SHORT	DETAIL
**Funding source**		
**Conflict of interest**		
**ROBINS-I assessment**		
**ROBINS-I Confounding**		
**ROBINS-I Selection into study**		
**ROBINS-I Classification of intervention**		
**ROBINS-I Deviation from intervention**		
**ROBINS-I Missing data**		
**ROBINS-I Measurement of outcomes**		
**ROBINS-I Selective reporting**		
**ROBINS-I Overall**		
**Cochrane RoB 2.0 assessment**		
**Cochrane - Randomization process**		
**Cochrane - Deviations from intended interventions**		
**Cochrane - Missing outcome data**		
**Cochrane - Measurement of the outcome**		
**Cochrane - Selection of the reported result**		
**Cochrane - Overall**		
**ROBINS-I & Cochrane:**<br>**Overall risk of bias** (high/low/unclear)		
**Other**		
**Comments**		
**Other**		

Supplement 3: List of excluded full-text papers

#	Title	Authors	Published Year	Journal	Volume	Issue	Pages	DOI
1	Immunogenicity and safety of the bivalent HPV vaccine in female patients with juvenile idiopathic arthritis: A prospective controlled observational cohort study	Heijstek M.W.; Scherpenisse M.; Groot N.; Tacke C.; Schepp R.M.; Buisman A.-M.; Berbers G.A.M.; Van Der Klis F.R.M.; Wulffraat N.M.	2014	Annals of the Rheumatic Diseases	73	8	1500-1507	10.1136/annrheumdis-2013-203429
2	Acute disseminated encephalomyelitis following immunization with human papillomavirus vaccines	Yoneda M.	2016	Internal Medicine	55	21	3077-3078	10.2169/internalmedicine.55.7217
3	Active surveillance for adverse events: The experience of the vaccine safety datalink project	Yih W.K.; Kulldorff M.; Fireman B.H.; Shui I.M.; Lewis E.M.; Klein N.P.; Baggs J.; Weintraub E.S.; Belongia E.A.; Naleway A.; Gee J.; Platt R.; Lieu T.A.	2011	Pediatrics	127	SUPPL. 1	S54-S64	10.1542/peds.2010-1722I
4	New quadrivalent HPV vaccine developments	Tovar J.M.; Bazaldua O.V.	2008	Postgraduate Medicine	120	4	14-16	10.3810/pgm.2008.11.1929
5	Safety of bivalent human papillomavirus vaccine in the US vaccine adverse event reporting system (VAERS), 2009â€“2017	Suragh T.A.; Lewis P.; Arana J.; Mba-Jonas A.; Li R.; Stewart B.; Shimabukuro T.T.; Cano M.	2018	British Journal of Clinical Pharmacology	84	12	2928-2932	10.1111/bcp.13736
6	Safety of a quadrivalent human papillomavirus (HPV) vaccine in patients with systemic lupus erythematosus	Mok C.C.; Chan P.T.; Ho L.Y.; Yu K.L.; To C.H.	2011	Arthritis and Rheumatism	63	10		
7	Acute disseminated encephalomyelitis with tumefactive lesions after vaccination against human papillomavirus	Mendoza Plasencia Z.; GonzÃ¡lez LÃ³pez M.; FernÃ¡ndez Sanfiel M.L.; MuÃ±iz Montes J.R.	2010	Neurologia	25	1	58-59	10.1016/S0213-4853(10)70023-2
8	Autoimmune disorders following HPV vaccination in young women: Is the risk real?	Grimaldi-Bensouda L.; Rossignol M.; Karam F.; Papo T.; Vermersh P.; Bourgault I.; Dachez R.; Breart G.; Abenhaim L.	2016	Pharmacoepidemiology and Drug Safety	25	(Breart G.) Inserm U149 UniversitÃ© Pierre et Marie	504	10.1002/pds.4070

						Curie, Paris, France		
9	Gardasil CNS demyelinating diseases		2009	Gynakologische Praxis	33	4	738-739	
10	HPV 6,11,16,18 vaccine: Pharmacovigilance reports in 2007		2008	Prescrire International	17	96	160	
11	Human papillomavirus vaccines and guillain-BarrÃ© syndrome: Managing uncertainties		2016	Prescrire International	25	176	265-270	
12	Human papillomavirus vaccines: 2014 Safety review		2015	Prescrire International	24	160	122-125 and 128-129	
13	IDdb News focus		2002	Current Drug Discovery		MAY	13-16	
14	MHRA safety review backs Cervarix		2013	Drug and Therapeutics Bulletin	51	2	16-17	
15	Risk of autoimmune diseases (AD) after human papillomavirus (HPV)-16/18 AS04-adjuvanted vaccine immunization in women aged 9 to 25 years in the United Kingdom: An observational cohort study	Willame C.; Rosillon D.; Zima J.; Angelo M.-G.; Stuurman A.; Vroling H.; Van Staa T.; Boggon R.; Bunge E.; Pladevall-Vila M.; Baril L.	2015	Pharmacoepidemiology and Drug Safety	24	(Pladevall-Vila M.) RTI Health Solutions, Barcelona, Spain	409	10.1002/pds.3838
16	Human papillomavirus vaccine and autoimmune disorders: A cohort study of 5.8 millions of adolescents and young women. Two years follow-up results	Tricotel A.; Fagot J.; Tubert-Bitter P.; Escolano S.; Weill A.; Castot A.	2011	Fundamental and Clinical Pharmacology	25	(Tricotel A.; Fagot J.; Tubert-Bitter P.; Escolano S.; Weill A.; Castot A.) Saint-Denis, France	101	10.1111/j.1472-8206.2011.00930.x
17	No autoimmune safety signal after vaccination with quadrivalent HPV vaccine Gardasil?	Tomljenovic L.; Shaw C.A.	2012	Journal of Internal Medicine	272	5	514-515	10.1111/j.1365-2796.2012.02551.x
18	Adverse effects of human papillomavirus		2012	Prescrire International	21	128	156-157	

	vaccines: Data available in 2011							
19	First reports of adverse drug reactions (ADRs) in recent weeks		2004	Drugs and Therapy Perspectives	20	10	20-22	10.2165/00042310-200420100-00007
20	Safety and immunogenicity of the quadrivalent HPV vaccine in girls with juvenile systemic lupus erythematosus and dermatomyositis	Pileggi G.S.; Pinto N.B.F.; Oliveira A.L.; Grein I.H.R.; Sztajnbok F.R.; Sato J.O.; Bicas B.; Almeida R.G.; Paim L.; Aikawa N.E.; Appenzeller S.; Fraga M.M.; Fraga A.C.; Barbosa C.M.P.L.; Dos Santos M.C.; IslabÃ£o A.; Robazzi T.C.M.V.; Bandeira M.; De Oliveira S.K.F.; MagalhÃ£es C.S.; Ferriani V.P.L.	2018	Advances in Rheumatolo gy	58	(MagalhÃ£ es C.S.) Faculdade de Medicina de BOTUCAT U-UNESP, Brazil		10.1186/s42358-018-0019-7
21	Using robust methods to assess the risk of serious adverse events following quadrivalent HPV vaccination	Levesque L.E.; Smith L.M.; Liu E.; Cheung M.; Lim W.T.; Lougheed D.; Ellis A.; Sears K.; Whitaker H.; Farrington C.P.	2015	Pharmacoep idemiology and Drug Safety	24	(Whitaker H.; Farrington C.P.) Mathemati cs and Statistics, Open University, Milton Keynes, United Kingdom	409-410	10.1002/pds.3838
22	Human papillomavirus vaccine [types 6, 11, 16, 18] (gardasil+) and autoimmune disorders: Safety assessment using the pharmacoepidemiologic general research extension system	Lamiae G.-B.; Rossignol M.; Aubrun E.; Leighton P.; Guillemot D.; Mahr A.; Benichou J.; Lambert P.-H.; Godeau B.; Abenhaim L.	2012	Arthritis and Rheumatism	64	(Abenhaim L.) LA-SER Europe Ltd, London, United Kingdom	S774	10.1002/art.37735
23	Vaccination with cevarix or gardasil	Knuf M.	2009	Gynakologis che Praxis	33	4	758-760	
24	Possible side effects from HPV vaccination in Denmark	Hammer A.; Petersen L.K.; Rolving N.; Boxill M.F.; KallesÃ¸e K.H.; Becker S.; Fredberg U.; SÃ¸rensen V.N.; Rask C.U.; Fink P.K.; BlaakÃ¦r J.	2016	Ugeskrift for laeger	178	26		
25	Complications of human papilloma virus vaccines	GÃ¼rsu T.; Desteli G.; Ayhan A.	2014	Turk Jinekolojik	17	3	79-83	

				Onkoloji Dergisi				
26	Comment: Human papillomavirus vaccination, induced autoimmunity, and neuromyelitis optica	Farrell R.A.	2012	Neurology	79	3	287	10.1212/WNL.0b013e31825fe10e
27	Autoimmune, neurologic, and venous thromboembolic adverse events following administration of a quadrivalent HPV vaccine to adolescent girls in Denmark and Sweden	Arnheim-DahlstrÃ¶m L.; Pasternak B.; SvanstrÃ¶m H.; SparÃ©n P.; Hviid A.	2013	Pharmacoepidemiology and Drug Safety	22	(Pasternak B.; SvanstrÃ¶m H.; Hviid A.) Department of Epidemiology Research, Statens Serum Institut, Copenhagen, Denmark	445	10.1002/pds.3512
28	Immunization with quadrivalent HPV vaccine (GARDASILÂ®) appears safe and induces antibody response in JIA: An interim analysis	Singer N.; Wagner-Weiner L.; Nanda K.; Robinson A.; Spalding S.; BÃ¼kÃ¼lmez H.	2014	Annals of the Rheumatic Diseases	73	(BÃ¼kÃ¼lmez H.) Pediatric Rheumatology, MetroHealth Medical Center and Case, Western Reserve University, Cleveland, United States		10.1136/annrheumdis-2014-eular.3050
29	Design and feasibility of a study using the clinical practice research datalink general practice online database (CPRD gold) to assess the risk of new onset of auto-immune diseases	Rosillon D.; Willame C.; Pladevall M.; Zima J.; Van Den Bosch J.H.; Bunge E.; Van Staa T.; Boggon R.; Baril L.	2014	Pharmacoepidemiology and Drug Safety	23	(Boggon R.) CPRD Research Group, London, United Kingdom	164-165	10.1002/pds.3701

	(NOAD) following administration of the human papillomavirus (HPV)-16/18 AS04-adjuvanted vaccine							
30	Sustained immunogenicity of the HPV-16/18 AS04-adjuvanted vaccine administered as a two-dose schedule in adolescent girls: Five-year clinical data and modeling predictions from a randomized study	Romanowski B.; Schwarz T.F.; Ferguson L.; Peters K.; Dionne M.; Behre U.; Schulze K.; Hillemanns P.; Suryakiran P.; Thomas F.; Struyf F.	2016	Human Vaccines and Immunother apeutics	12	1	20-29	10.1080/21645515.2015. 1065363
31	Immunogenicity and safety of the HPV-16/18 AS04-adjuvanted vaccine administered as a 2-dose schedule compared with the licensed 3-dose schedule: Results from a randomized study	Romanowski B.; Schwarz T.F.; Ferguson L.M.; Peters K.; Dionne M.; Schulze K.; Ramjattan B.; Hillemanns P.; Catteau G.; Dobbelaere K.; Schuind A.; Descamps D.	2011	Human Vaccines	7	12	1374-1386	10.4161/hv.7.12.18322
32	Questions remain about the human papillomavirus vaccine	Printz C.	2009	Cancer	115	4	699-701	10.1002/cncr.24080
33	2-Dose Schedule of AS04-Adjuvanted Human Papillomavirus Types 16/18 Vaccine	Poddighe D.	2017	Journal of Infectious Diseases	216	6	782-783	10.1093/infdis/jix364
34	Human papillomavirus vaccine in patients with systemic lupus erythematosus	Pellegrino P.; Carnovale C.; Perrone V.; Salvati D.; Gentili M.; Antoniazzi S.; Clementi E.; Radice S.	2014	Epidemiolog y	25	1	155-156	10.1097/EDE.000000000 0000033
35	Postlicensure safety evaluation of human papilloma virus vaccines	Labadie J.	2011	International Journal of Risk and Safety in Medicine	23	2	103-112	10.3233/JRS-2011-0529
36	The HPV vaccination crisis in Japan	Hanley S.J.B.	2016	Acta Cytologica	60	(Hanley S.J.B.) Departmen t of Women's	36	10.1159/000446388

						Health Medicine, Hokkaido University Graduate School of Medicine, Japan		
37	Authorsâ€™ response: Letter to the Editor â€“ HPV vaccine and autoimmunity	GrÃ¶nlund O.; Herweijer E.; SundstrÃ¶m K.; Arnheim-DahlstrÃ¶m L.	2017	Journal of Internal Medicine	281	3	311-312	10.1111/joim.12574
38	Quadrivalent HPV vaccination was not linked to multiple sclerosis or other demyelinating diseases	Granwehr B.	2015	Annals of Internal Medicine	162	8	JC13	
39	Safety of gardasilÂ® vaccine in systemic lupus erythematosus	Dhar J.P.; Essenmacher L.; Dhar R.; Magee A.; Ager J.; Venkatram M.; Sagar H.; Sokol R.	2013	Arthritis and Rheumatism	65	(Dhar J.P.; Essenmacher L.; Dhar R.; Magee A.; Ager J.; Venkatram M.; Sagar H.; Sokol R.) Wayne State University, Detroit, MI, United States	S1214-S1215	10.1002/art.38216
40	Adverse events and quadrivalent human papillomavirus recombinant vaccine	Debold V.; Hurwitz E.	2009	JAMA - Journal of the American Medical Association	302	24	2657	10.1001/jama.2009.1880
41	Letter to the editor â€“ HPV vaccine and autoimmunity Incidence of new-onset autoimmune disease in girls and women with pre-existing autoimmune disease after	Dahan S.; Shoenfeld Y.	2017	Journal of Internal Medicine	281	3	313-315	10.1111/joim.12575

	quadrivalent human papillomavirus vaccination: a cohort study							
42	Response to letter to editor: HPV vaccines and autoimmune diseases	Chao C.; Jacobsen S.J.	2012	Journal of Internal Medicine	272	1	99	10.1111/j.1365-2796.2012.02544.x
43	Evaluation of autoimmune safety signal in observational vaccine safety studies	Chao C.; Jacobsen S.	2012	Human Vaccines and Immunother apeutics	8	9	1302-1304	10.4161/hv.21268
44	Human papillomavirus vaccination and risk of autoimmune diseases: A large cohort study of over 2 million young girls in France	Chaignot C.; Miranda S.; Collin C.; Dray-Spira R.; Weill A.; Zureik M.	2016	Pharmacoep idemiology and Drug Safety	25	(Miranda S.; Collin C.; Dray-Spira R.; Zureik M.) French National Agency for Medicines and Health Products Safety (ANSM), Paris, France	473	10.1002/pds.4070
45	No association of HPV vaccination with serious adverse events	Caskey R.	2014	Journal of Pediatrics	164	5	1240	10.1016/j.jpeds.2014.02.0 43
46	Safety of the quadrivalent human papillomavirus vaccine: Now well established	Brotherton J.M.L.	2013	BMJ (Online)	347	7930		10.1136/bmj.f5631
47	Post-licensure safety monitoring of quadrivalent human papillomavirus vaccine in the Vaccine Adverse Event Reporting System (VAERS), 2009â€“2015	Arana J.E.; Harrington T.; Cano M.; Lewis P.; Mba-Jonas A.; Rongxia L.; Stewart B.; Markowitz L.E.; Shimabukuro T.T.	2018	Vaccine	36	13	1781-1788	10.1016/j.vaccine.2018.0 2.034
48	Safety and immunogenicity of the quadrivalent HPV	Soybilgic A.; Onel K.B.; Utset T.; Alexander K.; Wagner-Weiner L.	2013	Pediatric Rheumatolo gy	11	1		10.1186/1546-0096-11-29

	vaccine in female Systemic Lupus Erythematosus patients aged 12 to 26 years							
49	Postlicensure safety surveillance for quadrivalent human papillomavirus recombinant vaccine	Slade B.A.; Leidel L.; Vellozzi C.; Woo E.J.; Hua W.; Sutherland A.; Izurieta H.S.; Ball R.; Miller N.; Braun M.M.; Markowitz L.E.; Iskander J.	2009	Obstetrical and Gynecologic al Survey	64	12	796-798	10.1097/01.ogx.0000363 241.72396.31
50	HPV vaccines and autoimmune diseases	Shoenfeld Y.	2012	Journal of Internal Medicine	272	1	98	10.1111/j.1365-2796.2012.02537.x
51	Sustained immunogenicity and efficacy of the HPV-16/18 AS04-adjuvanted vaccine: Up to 8.4 years of follow-up	Roteli-Martins C.M.; Naud P.; De Borba P.; Teixeira J.C.; De Carvalho N.S.; Zahaf T.; Sanchez N.; Geeraerts B.; Descamps D.	2012	Human Vaccines and Immunother apeutics	8	3	381-388	10.4161/hv.8.3.18865
52	Safety and Immunogenicity of the HPV-16/18 AS04-Adjuvanted Vaccine: A Randomized, Controlled Trial in Adolescent Girls	Rivera Medina D.M.; Valencia A.; de Velasquez A.; Huang L.-M.; Prymula R.; GarcÃa-Sicilia J.; Rombo L.; David M.P.P.; Descamps D.; Hardt K.; Dubin G.	2010	Journal of Adolescent Health	46	5	414-421	10.1016/j.jadohealth.2010 .02.006
53	Efficacy of a prophylactic adjuvanted bivalent L1 virus-like-particle vaccine against infection with human papillomavirus types 16 and 18 in young women: an interim analysis of a phase III double-blind, randomised controlled trial	Paavonen J.; Jenkins D.; Bosch F.X.; Naud P.; SalmerÃ³n J.; Wheeler C.M.; Chow S.-N.; Apter D.L.; Kitchener H.C.; Castellsague X.; de Carvalho N.S.; Skinner S.R.; Harper D.M.; Hedrick J.A.; Jaisamrarn U.; Limson G.A.; Dionne M.; Quint W.; Spiessens B.; Peeters P.; Struyf F.; Wieting S.L.; Lehtinen M.O.; Dubin G.	2007	Lancet	369	9580	2161-2170	10.1016/S0140-6736(07)60946-5
54	Immunogenicity and safety of a quadrivalent human papillomavirus vaccine in patients with systemic lupus erythematosus: A case-control study	Mok C.C.; Ho L.Y.; Fong L.S.; To C.H.	2013	Annals of the Rheumatic Diseases	72	5	659-664	10.1136/annrheumdis-2012-201393
55	Quadrivalent human papillomavirus vaccination in girls and	Liu E.Y.; Smith L.M.; Ellis A.K.; Whitaker H.; Law B.; Kwong J.C.; Farrington P.; LÃ©vesque L.E.	2018	CMAJ	190	21	E648-E655	10.1503/cmaj.170871

	the risk of autoimmune disorders: The Ontario Grade 8 HPV Vaccine Cohort Study							
56	Comparative immunogenicity and safety of human papillomavirus (HPV)-16/18 AS04-adjuvanted vaccine and HPV-6/11/16/18 vaccine administered according to 2- and 3-dose schedules in girls aged 9â€“14 years: Results to month 12 from a randomized trial	Leung T.F.; Liu A.P.-Y.; Lim F.S.; Thollot F.; Oh H.M.L.; Lee B.W.; Rombo L.; Tan N.C.; Rouzier R.; Friel D.; de Muynck B.; de Simoni S.; Suryakiran P.; Hezareh M.; Folschweiller N.; Thomas F.; Struyf F.	2015	Human Vaccines and Immunotherapeutics	11	7	1689-1702	10.1080/21645515.2015.1050570
57	Overall efficacy of HPV-16/18 AS04-adjuvanted vaccine against grade 3 or greater cervical intraepithelial neoplasia: 4-year end-of-study analysis of the randomised, double-blind PATRICIA trial	Lehtinen M.; Paavonen J.; Wheeler C.M.; Jaisamrarn U.; Garland S.M.; CastellsaguÃ© X.; Skinner S.R.; Apter D.; Naud P.; SalmerÃ³n J.; Chow S.-N.; Kitchener H.; Teixeira J.C.; Hedrick J.; Limson G.; Szarewski A.; Romanowski B.; Aoki F.Y.; Schwarz T.F.; Poppe W.A.J.; De Carvalho N.S.; Germar M.J.V.; Peters K.; Mindel A.; De Sutter P.; Bosch F.X.; David M.-P.; Descamps D.; Struyf F.; Dubin G.	2012	The Lancet Oncology	13	1	89-99	10.1016/S1470-2045(11)70286-8
58	Editorial	KÃ¼nzel W.; Drife J.	2009	European Journal of Obstetrics Gynecology and Reproductive Biology	145	2	127-128	10.1016/j.ejogrb.2009.06.019
59	HPV vaccine, is it really harmful?	Kim S.	2014	Journal of Korean medical science	29	6	749-750	10.3346/jkms.2014.29.6.749
60	Response to: 'Bivalent HPV vaccine safety depending on subtypes of juvenile idiopathic arthritis' by Dr Akioka	Heijstek M.W.; Wulffraat N.M.	2014	Annals of the Rheumatic Diseases	73	12	e76-e76	10.1136/annrheumdis-2014-206426

61	Safety and immunogenicity of human papillomavirus vaccination in juvenile patients with rheumatic diseases	Heijstek M.W.; Groot N.; Scherpenisse M.; Tacke C.; Berbers G.; Van Der Klis F.; Wulffraat N.M.	2011	Pediatric Rheumatology	9	(Scherpenisse M.; Van Der Klis F.) Centre for Infectious Disease Control Netherlands, National Institute of Public Health and the Environment, Bilthoven, Netherlands		
62	Response to: HPV vaccine and autoimmunity Incidence of new-onset autoimmune disease in girls and women with pre-existing autoimmune disease after quadrivalent human papillomavirus vaccination: a cohort study	Hawkes D.; Wilkinson W.; Dunlop R.A.	2017	Journal of Internal Medicine	281	5	530-531	10.1111/joim.12602
63	The risks and benefits of HPV vaccination	Haug C.	2009	JAMA - Journal of the American Medical Association	302	7	795-796	10.1001/jama.2009.1215
64	Authorsâ€² Reply	Gupta S.; Kerkar R.; Dikshit R.; Badwe R.	2014	South Asian Journal of Cancer	3	1	94-95	10.4103/2278-330X.126580
65	Autoimmune disorders and quadrivalent human papillomavirus vaccination of young females	Grimaldi-Bensouda L.; Guillemot D.; Godeau B.; Mahr A.; Lambert P.-H.; Benichou J.; Lebrun-Frenay C.; Papeix C.; Labauge P.; Berquin P.; Penfornis A.; Benhamou P.-Y.; Nicolino M.; Simon A.; Viallard J.-F.; Costedoat-	2013	Pharmacoepidemiology and Drug Safety	22	(Abenhaim L.) LA-SER Europe Limited, London,	203-204	10.1002/pds.3512

		Chalumeau N.; Courcoux M.-F.; Pondarre C.; Hilliquin P.; Chatelus E.; Foltz V.; Guillaume S.; Rossignol M.; Abenhaim L.				United Kingdom		
66	Vaccine-associated guillain-barre syndrome: A pharmacovigilance analysis of data in the United States' vaccine adverse event reporting system (1990-2009)	Ali A.K.	2011	Value in Health	14	3	A113-A114	
67	Post-marketing study of autoimmune diseases following vaccination with human papillomavirus bivalent (types 16 and 18) recombinant vaccine in females in the US: Rationale and design	Galindo C.M.; Rosillon D.; Holick C.N.; Rodgers K.; Quinlan S.; Adler J.; Buyse H.; Verstraeten T.	2011	Pharmacoepidemiology and Drug Safety	20	(Holick C.N.; Rodgers K.; Quinlan S.) Health Core Inc., Wilmington, DE, United States	S359	10.1002/pds.2206
68	Immunogenicity, safety and tolerability of a bivalent human papillomavirus vaccine in adolescents with juvenile idiopathic arthritis	Esposito S.; Corona F.; Barzon L.; Cuoco F.; Squarzon L.; Marcati G.; Torcoletti M.; Gambino M.; PalÃ¹ G.; Principi N.	2014	Expert Review of Vaccines	13	11	1387-1393	10.1586/14760584.2014.943195
69	Letter from the editor	Ellis R.; Weiss A.	2016	Human Vaccines and Immunotherapeutics	12	3	557	10.1080/21645515.2016.1160702
70	Letter from the editor	Ellis R.; Riedmann E.M.	2014	Human Vaccines and Immunotherapeutics	10	1	2677-2678	10.4161/hv.28050
71	Development of clinically isolated syndrome after vaccination, a CDC/FDA vaccine adverse event reporting system (VAERS) study, [1985-2017]	Eddin M.F.; Patel J.; Alchaki A.R.; Souayah N.	2018	Neurology	90	15		

72	The safety and immunogenicity of Quadrivalent HPV (qHPV) vaccine in systemic lupus erythematosus	Dhar J.P.; Essenmacher L.; Dhar R.; Magee A.; Ager J.; Sokol R.J.	2017	Vaccine	35	20	2642-2646	10.1016/j.vaccine.2017.04.001
73	Safety of human papillomavirus (HPV)-16/18 AS04-adjuvanted vaccine for cervical cancer prevention: A pooled analysis of 11 clinical trials	Descamps D.; Hardt K.; Spiessens B.; Izurieta P.; Verstraeten T.; Breuer T.; Dubin G.	2009	Human Vaccines	5	5	332-340	
74	Quadrivalent human papillomavirus (types 6, 11, 16, 18) recombinant vaccine	Cada D.J.; Levien T.; Baker D.E.	2006	Hospital Pharmacy	41	12	1185-1192	
75	Pooled analysis of large and long-term safety data from the human papillomavirus-16/18-AS04-adjuvanted vaccine clinical trial programme	Angelo M.-G.; David M.-P.; Zima J.; Baril L.; Dubin G.; Arellano F.; Struyf F.	2014	Pharmacoepidemiology and Drug Safety	23	5	466-479	10.1002/pds.3554
76	Human papillomavirus immunisation of adolescent girls and anticipated reporting of immune-mediated adverse events	CallrÃ©us T.; SvanstrÃ¶m H.; Nielsen N.M.; Poulsen S.; Valentiner-Branth P.; Hviid A.	2009	Vaccine	27	22	2954-2958	10.1016/j.vaccine.2009.02.106
77	Autoimmune, neurological, and venous thromboembolic adverse events after immunisation of adolescent girls with quadrivalent human papillomavirus vaccine in Denmark and Sweden: Cohort study	Arnheim-DahlstrÃ¶m L.; Pasternak B.; SvanstrÃ¶m H.; SparÃ©n P.; Hviid A.	2013	BMJ (Online)	347	7930		10.1136/bmj.f5906
78	Bivalent HPV vaccine safety depending on subtypes of juvenile idiopathic arthritis	Akioka S.	2014	Annals of the Rheumatic Diseases	73	12	e75	10.1136/annrheumdis-2014-206426

79	An Overview of Quadrivalent Human Papillomavirus Vaccine Safety: 2006 to 2015	Vichnin M.; Bonanni P.; Klein N.P.; Garland S.M.; Block S.L.; Kjaer S.K.; Sings H.L.; Perez G.; Haupt R.M.; Saah A.J.; Lievano F.; Velicer C.; Drury R.; Kuter B.J.	2015	Pediatric Infectious Disease Journal	34	9	983-991	10.1097/INF.0000000000000793
80	Human papillomavirus (HPV) vaccines as an option for preventing cervical malignancies: (How) effective and safe?	Tomljenovic L.; Spinosa J.P.; Shaw C.A.	2013	Current Pharmaceutical Design	19	8	1466-1487	
81	Safety of human papillomavirus vaccines: A review	Stillo M.; Carrillo Santisteve P.; Lopalco P.L.	2015	Expert Opinion on Drug Safety	14	5	697-712	10.1517/14740338.2015.1013532
82	Current global status & impact of human papillomavirus vaccination: Implications for India	Sankaranarayanan R.; Bhatla N.; Basu P.	2016	Indian Journal of Medical Research	144	August	169-180	10.4103/0971-5916.195023
83	Guillain-BarrÃ© Syndrome and Immunizations	Randall D.P.	2010	Disease-a-Month	56	5	293-298	10.1016/j.disamonth.2010.02.009
84	Vaccine-preventable diseases, vaccines and Guillain-Barreâ€™ syndrome	Principi N.; Esposito S.	2018	Vaccine		(Esposito S., susanna.esposito@unimi.it) Pediatric Clinic, Department of Surgical and Biomedical Sciences, UniversitÃ degli Studi di Perugia, Perugia, Italy		10.1016/j.vaccine.2018.05.119
85	Quadrivalent human papillomavirus (HPV) vaccine: A review of safety, efficacy, and pharmacoeconomics	Pomfret T.C.; Gagnon Jr. J.M.; Gilchrist A.T.	2011	Journal of Clinical Pharmacy and Therapeutics	36	1	01-Sep	10.1111/j.1365-2710.2009.01150.x

86	Safety of Human Papillomavirus Vaccines: An Updated Review	Phillips A.; Patel C.; Pillsbury A.; Brotherton J.; Macartney K.	2018	Drug Safety	41	4	329-346	10.1007/s40264-017-0625-z
87	On the relationship between human papilloma virus vaccine and autoimmune diseases	Pellegrino P.; Carnovale C.; Pozzi M.; Antoniazzi S.; Perrone V.; Salvati D.; Gentili M.; Brusadelli T.; Clementi E.; Radice S.	2014	Autoimmunit y Reviews	13	7	736-741	10.1016/j.autrev.2014.01.054
88	Immunogenicity and safety of the human papillomavirus vaccine in patients with autoimmune diseases: A systematic review	Pellegrino P.; Radice S.; Clementi E.	2015	Vaccine	33	30	3444-3449	10.1016/j.vaccine.2015.05.041
89	Vaccines and autoimmune diseases of the adult.	Orbach H.; Agmon-Levin N.; Zandman-Goddard G.	2010	Discovery medicine	9	45	90-97	
90	Safety of quadrivalent human papillomavirus vaccine	Omer S.B.	2012	Journal of Internal Medicine	271	2	177-178	10.1111/j.1365-2796.2011.02481.x
91	Anti-HPV vaccines: they are proven to be safe and do not provoke autoimmune diseases	Nau J.-Y.	2015	Revue mÃ©dicale suisse	11	487	1770-1771	
92	Causal relationship between immunological responses and adverse reactions following vaccination	Nakayama T.	2019	Vaccine	37	2	366-371	10.1016/j.vaccine.2018.11.045
93	Human papillomavirus vaccine and demyelinating diseasesâ€"A systematic review and meta-analysis	Mouchet J.; Salvo F.; Raschi E.; Poluzzi E.; Antonazzo I.C.; De Ponti F.; BÃ©gaud B.	2018	Pharmacolo gical Research	132	(Raschi E.; Poluzzi E.; Antonazzo I.C.; De Ponti F.) Departmen t of Medical and Surgical Sciences, University of Bologna, Bologna, Italy	108-118	10.1016/j.phrs.2018.04.007

94	Safety of human papillomavirus vaccines: A review	Macartney K.K.; Chiu C.; Georgousakis M.; Brotherton J.M.L.	2013	Drug Safety	36	6	393-412	10.1007/s40264-013-0039-5
95	CDC panel recommends vaccine for smokers; reviews HPV safety data	Kuehn B.M.	2008	JAMA - Journal of the American Medical Association	300	23	2713-2714	10.1001/jama.2008.776
96	Safety and efficacy data on vaccines and immunization to human papillomavirus	Kash N.; Lee M.A.; Kollipara R.; Downing C.; Guidry J.; Tyring S.K.	2015	Journal of Clinical Medicine	4	4	614-633	10.3390/jcm4040614
97	Answering human papillomavirus vaccine concerns; A matter of science and time	Hawkes D.; Lea C.E.; Berryman M.J.	2013	Infectious Agents and Cancer	8	1		10.1186/1750-9378-8-22
98	Prophylactic human papillomavirus vaccines to prevent cervical cancer: Review of the Phase II and III trials	Harper D.M.	2008	Therapy	5	3	313-324	10.2217/14750708.5.3.313
99	HPV vaccine and autoimmune diseases: systematic review and meta-analysis of the literature	Genovese C.; LA Fauci V.; Squeri A.; Trimarchi G.; Squeri R.	2018	Journal of preventive medicine and hygiene	59	3	E194-E199	10.15167/2421-4248/jpmh2018.59.3.998
100	Quadrivalent HPV vaccine safety review and safety monitoring plans for nine-valent HPV vaccine in the United States	Gee J.; Weinbaum C.; Sukumaran L.; Markowitz L.E.	2016	Human Vaccines and Immunotherapeutics	12	6	1406-1417	10.1080/21645515.2016.1168952
101	Primary Prevention of HPV through Vaccination: Update on the Current Global Status	Brotherton J.M.L.; Zuber P.L.F.; Bloem P.J.N.	2016	Current Obstetrics and Gynecology Reports	5	3	210-224	10.1007/s13669-016-0165-z
102	Human papillomavirus vaccination: Where are we now?	Brotherton J.M.L.	2014	Journal of Paediatrics and Child Health	50	12	959-965	10.1111/jpc.12627
103	A summary of the post-licensure surveillance	Bonanni P.; Cohet C.; Kjaer S.K.; Latham N.B.; Lambert P.-H.; Reisinger K.; Haupt R.M.	2010	Vaccine	28	30	4719-4730	10.1016/j.vaccine.2010.04.070

	initiatives for GARDASIL/SILGARDÂ®							
104	Post-licensure safety surveillance for human papillomavirus-16/18-AS04-adjuvanted vaccine: More than 4years of experience	Angelo M.-G.; Zima J.; Tavares Da Silva F.; Baril L.; Arellano F.	2014	Pharmacoepidemiology and Drug Safety	23	5	456-465	10.1002/pds.3593
105	Safety of human papillomavirus (HPV) vaccines: A review of the international experience so far	Agorastos T.; Chatzigeorgiou K.; Brotherton J.M.L.; Garland S.M.	2009	Vaccine	27	52	7270-7281	10.1016/j.vaccine.2009.09.097
106	Human papillomavirus vaccine safety in Australia: Experience to date and issues for surveillance	Gold M.S.; Buttery J.; McIntyre P.	2010	Sexual Health	7	3	320-324	10.1071/SH09153

Updated search: full-text exclusions

#	Title	Authors	Published Year	Journal	Volume	Issue	Pages	DOI
1	Do Vaccines Trigger Neurological Diseases? Epidemiological Evaluation of Vaccination and Neurological Diseases Using Examples of Multiple Sclerosis, Guillain–Barré Syndrome and Narcolepsy	Stowe J., Andrews N., Miller E.	2020	CNS Drugs	34	1	1-8	10.1007/s40263-019-00670-y.
2	Efficacy, immunogenicity and safety of the AS04-HPV-16/18 vaccine in Chinese women aged 18-25 years: End-of-study results from a phase II/III, randomised, controlled trial	Zhu F.-C., Hu S.-Y., Hong Y., Hu Y.-M., Zhang X., Zhang Y.-J., Pan Q.-J., Zhang W.-H., Zhao F.-H., Zhang C.-F., Yang X., Yu J.-X., Zhu J., Zhu Y., Chen F., Zhang Q., Wang H., Wang C., Bi J., Xue S., Shen L., Zhang Y.-S., He Y., Tang H., Karkada N., Suryakiran P., Bi D., Struyf F.	2019	Cancer Medicine	8	14	6195-6211	10.1002/cam4.2399
3	Safety profile of human papilloma virus vaccines:	Bonaldo G., Vaccheri A., D'Annibali O., Motola D.	2019	British Journal of	85	3	634-643	10.1111/bcp.13841

	an analysis of the US Vaccine Adverse Event Reporting System from 2007 to 2017			Clinical Pharmacology				
4	Adverse events following Quadrivalent HPV vaccination reported in Sao Paulo State, Brazil, in the first three years after introducing the vaccine for routine immunization (March 2014 to December 2016)	Mauro A.B., Fernandes E.G., Miyaji K.T., Arantes B.A., Valente M.G., Sato H.K., Sartori A.M.C.<br>Revista do Instituto de Medicina	2019	Tropical de Sao Paulo	61	E43		10.1590/S1678-9946201961043
5	Safety of bivalent human papillomavirus vaccine in the US vaccine adverse event reporting system (VAERS), 2009–2017	Suragh T.A., Lewis P., Arana J., Mba-Jonas A., Li R., Stewart B., Shimabukuro T.T., Cano M.	2018	British Journal of Clinical Pharmacology	84	12	2928	10.1111/bcp.13736
6	Quadrivalent human papillomavirus vaccination in girls and the risk of autoimmune disorders: The Ontario Grade 8 HPV Vaccine Cohort Study	Liu E.Y., Smith L.M., Ellis A.K., Whitaker H., Law B., Kwong J.C., Farrington P., Lévesque L.E.	2018	CMAJ	190	21	E648-E655	10.1503/cmaj.170871

Supplement 4: Risk of bias assessment

Supplement Table. Risk of bias assessment in randomized and non-randomized studies.

A) Risk of bias in randomized controlled trials (1)

Study	Randomization process	Deviations from intended interventions	Missing outcome data	Measurement of the outcome	Selection of the reported result	Overall
Lehtinen, 2016 (2) & Bi, 2019 (3)	Low (+)	Low (+)	Low (+)	High (-)	Low (+)	Hight (-)
Verstraeten, 2008 (4)	Unknown (?)	Unknown (?)	Unknown (?)	Unknown (?)	Unknown (?)	Unknown (?)

Legend: + low risk of bias; +/- some concerns; − high risk of bias; ? unknown risk of bias.

B) Risk of bias in non-randomized studies (5)

Study	Confounding	Selection bias	Classification of intervention	Deviation from intervention	Missing data	Outcome measurement	Selection of reported results	Overall risk of bias
Deceuninck, 2018 (6)	Critical (--)	Low (++)	Critical (--)	Low (++)	Low (++)	Serious (-)	Low (++)	Critical (--)
Chao, 2012 (7)	Critical (--)	Moderate (+)	Moderate (+)	Low (++)	Low (++)	Serious (-)	Low (++)	Critical (--)
Gee, 2011 (8)	Critical (--)	Low (++)	Moderate (+)	Low (++)	Low (++)	Low (++)	Low (++)	Critical (--)
Gee, 2017 (9)	Critical (--)	Low (++)	Moderate (+)	Low (++)	Low (++)	Serious (-)	Low (++)	Critical (--)
Donahue, 2019 (10)	Critical (--)	Low (++)	Moderate (+)	Low (++)	Low (++)	Serious (-)	Low (++)	Critical (--)
Slade, 2009 (11)	Critical (--)	Low (++)	Moderate (+)	Low (++)	Low (++)	Low (++)	Low (++)	Critical (--)
Souayah, 2011 (12)	Critical (--)	Low (++)	Moderate (+)	Low (++)	Low (++)	Serious (-)	Low (++)	Critical (--)
Geier, 2015 (13)	Critical (--)	Low (++)	Moderate (+)	Low (++)	Low (++)	Serious (-)	Low (++)	Critical (--)
Arana, 2018 (14)	Critical (--)	Low (++)	Moderate (+)	Low (++)	Low (++)	Low (++)	Low (++)	Critical (--)
Neha, 2020 (15)	Critical (--)	Low (++)	Moderate (+)	Low (++)	Low (++)	Low (++)	Low (++)	Critical (--)
Ojha, 2014 (16)	Moderate (+)	Low (++)	Moderate (+)	Low (++)	Low (++)	Serious (-)	Low (++)	Serious (-)
Geier, 2017 (17)	Critical (--)	Low (++)	Moderate (+)	Low (++)	Low (++)	Serious (-)	Low (++)	Critical (--)
Frisch, 2018 (18)	Moderate (+)	Low (++)	Moderate (+)	Low (++)	Low (++)	Serious (-)	Low (++)	Critical (--)
Skufca, 2018 (19)	Moderate (+)	Low (++)	Moderate (+)	Low (++)	Low (++)	Serious (-)	Low (++)	Serious (-)
Grönlund, 2016 (20)	Moderate (+)	Low (++)	Moderate (+)	Low (++)	Low (++)	Serious (-)	Low (++)	Serious (-)
Hviid, 2018 (21)	Critical (--)	Low (++)	Moderate (+)	Low (++)	Low (++)	Serious (-)	Low (++)	Critical (--)
Grimaldi-Bensouda, 2014 (22)	Critical (--)	Low (++)	Moderate (+)	Low (++)	Low (++)	Low (++)	Low (++)	Critical (--)
Grimaldi-Bensouda, 2017 (23)	Moderate (+)	Low (++)	Moderate (+)	Low (++)	Low (++)	Serious (-)	Low (++)	Serious (-)
Miranda, 2017 (24)	Moderate (+)	Low (++)	Moderate (+)	Low (++)	Low (++)	Serious (-)	Low (++)	Serious (-)
Andrews, 2017 (25)	Moderate (+)	Moderate (+)	Moderate (+)	Low (++)	Low (++)	Serious (-)	Low (++)	Serious (-)
Cameron, 2016 (26)	Critical (--)	Low (++)	Critical (--)	Low (++)	Low (++)	Serious (-)	Low (++)	Critical (--)
Willame, 2016 (27)	Moderate (+)	Low (++)	Moderate (+)	Low (++)	Low (++)	Serious (-)	Low (++)	Serious (-)

Legend: ++ low risk of bias; + moderate risk of bias; − serious risk of bias; – –- critical risk of bias.

Supplement 5: Meta-analysis of studies reporting an effect estimate of the risk of Guillain-Barré Syndrome after HPV-vaccination, by vaccination.

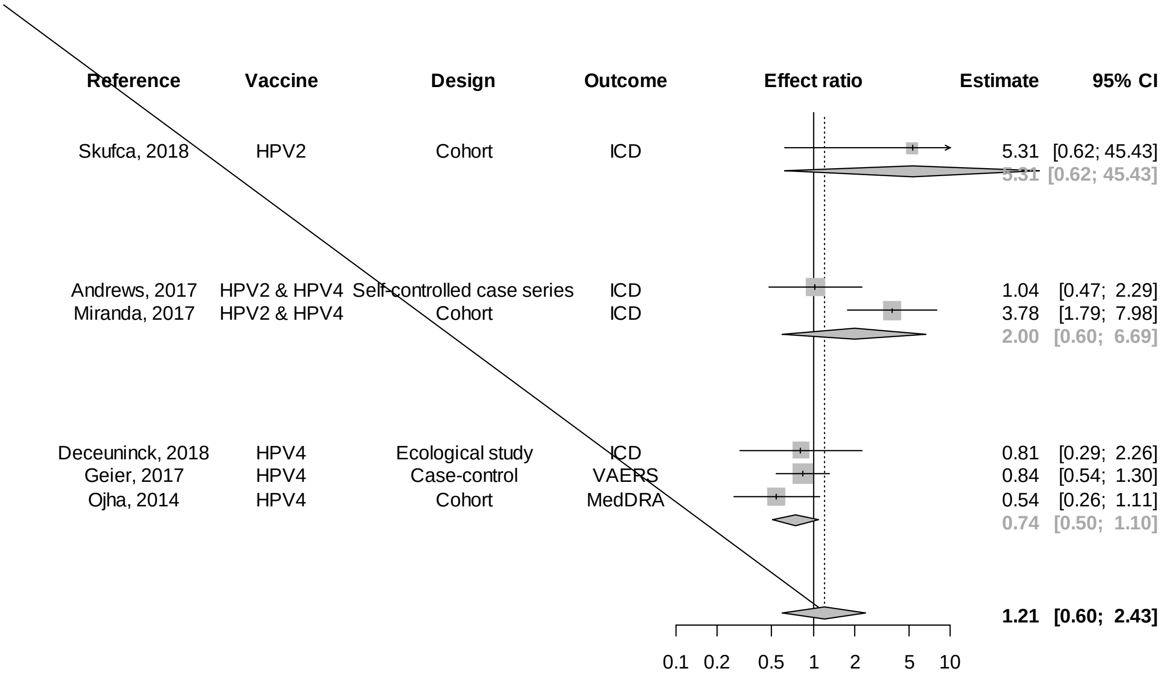

Supplement 6: Meta-analysis of studies reporting an effect estimate of the risk of Guillain-Barré Syndrome after HPV-vaccination, by outcome measurement.

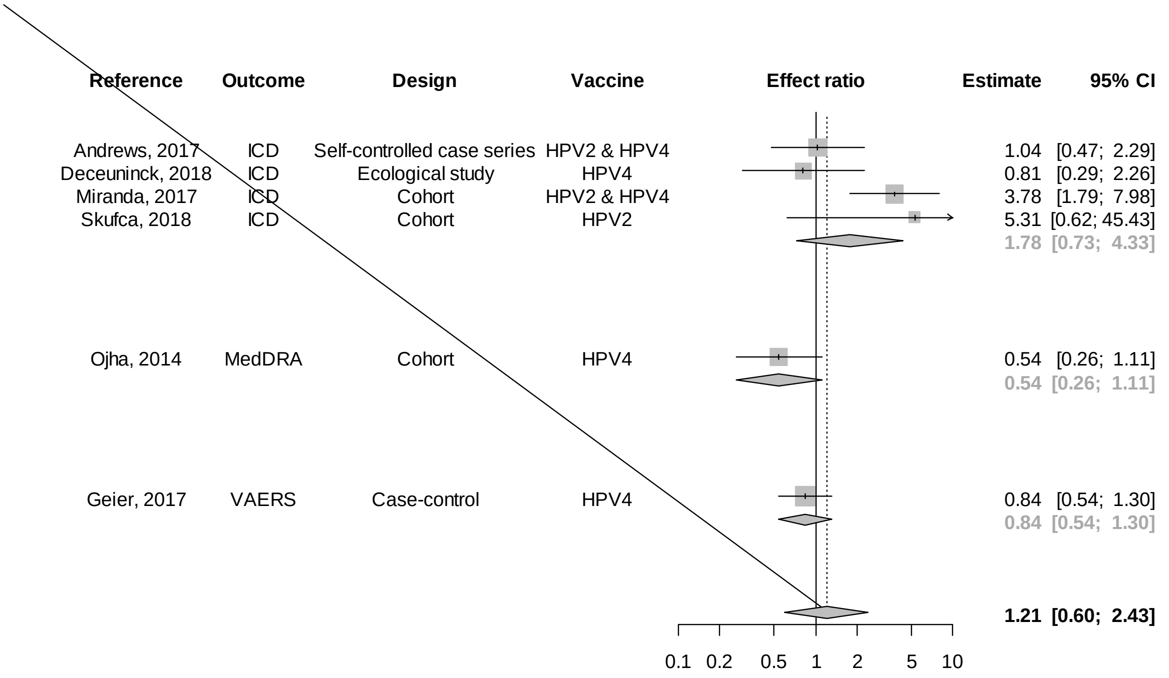

References

1. Sterne JAC, Savovic J, Page MJ, Elbers RG, Blencowe NS, Boutron I, et al. RoB 2: a revised tool for assessing risk of bias in randomised trials. BMJ. 2019;366:l4898.
2. Lehtinen M, Eriksson T, Apter D, Hokkanen M, Natunen K, Paavonen J, et al. Safety of the human papillomavirus (HPV)-16/18 AS04-adjuvanted vaccine in adolescents aged 12-15 years: Interim analysis of a large community-randomized controlled trial. Hum Vaccin Immunother. 2016;12(12):3177-85.
3. Bi D, Apter D, Eriksson T, Hokkanen M, Zima J, Damaso S, et al. Safety of the AS04-adjuvanted human papillomavirus (HPV)-16/18 vaccine in adolescents aged 12-15 years: end-of-study results from a community-randomized study up to 6.5 years. Hum Vaccin Immunother. 2019:1-12.
4. Verstraeten T, Descamps D, David MP, Zahaf T, Hardt K, Izurieta P, et al. Analysis of adverse events of potential autoimmune aetiology in a large integrated safety database of AS04 adjuvanted vaccines. Vaccine. 2008;26(51):6630-8.
5. Sterne JA, Hernan MA, Reeves BC, Savovic J, Berkman ND, Viswanathan M, et al. ROBINS-I: a tool for assessing risk of bias in non-randomised studies of interventions. BMJ. 2016;355:i4919.
6. Deceuninck G, Sauvageau C, Gilca V, Boulianne N, De Serres G. Absence of association between Guillain-Barré syndrome hospitalizations and HPV-vaccine. Expert Review of Vaccines. 2018;17(1):99-102.
7. Chao C, Klein NP, Velicer CM, Sy LS, Slezak JM, Takhar H, et al. Surveillance of autoimmune conditions following routine use of quadrivalent human papillomavirus vaccine. Journal of Internal Medicine. 2012;271(2):193-203.
8. Gee J, Naleway A, Shui I, Baggs J, Yin R, Li R, et al. Monitoring the safety of quadrivalent human papillomavirus vaccine: Findings from the Vaccine Safety Datalink. Vaccine. 2011;29(46):8279-84.
9. Gee J, Sukumaran L, Weintraub E. Risk of Guillain-Barré Syndrome following quadrivalent human papillomavirus vaccine in the Vaccine Safety Datalink. Vaccine. 2017;35(43):5756-8.
10. Donahue JG, Kieke BA, Lewis EM, Weintraub ES, Hanson KE, McClure DL, et al. Near Real-Time Surveillance to Assess the Safety of the 9-Valent Human Papillomavirus Vaccine. Pediatrics. 2019;144(6).
11. Slade BA, Leidel L, Vellozzi C, Woo EJ, Hua W, Sutherland A, et al. Postlicensure safety surveillance for quadrivalent human papillomavirus recombinant vaccine. Obstetrical and Gynecological Survey. 2009;64(12):796-8.
12. Souayah N, Michas-Martin PA, Nasar A, Krivitskaya N, Yacoub HA, Khan H, et al. Guillain-Barré syndrome after Gardasil vaccination: Data from Vaccine Adverse Event Reporting System 2006-2009. Vaccine. 2011;29(5):886-9.
13. Geier DA, Geier MR. Quadrivalent human papillomavirus vaccine and autoimmune adverse events: a case–control assessment of the vaccine adverse event reporting system (VAERS) database. Immunologic Research. 2017;65(1):46-54.
14. Arana J, Su J, Lewis P, Cano M, Markowitz LE, Shimabukuro T. 2463. Post-licensure Surveillance of 9-Valent Human Papillomavirus Vaccine (9vHPV) in the Vaccine Adverse Event Reporting System (VAERS), United States, 2014–2017. Open Forum Infectious Diseases. 2018;5(suppl_1):S738-S.
15. Neha R, Subeesh V, Beulah E, Gouri N, Maheswari E. Postlicensure surveillance of human papillomavirus vaccine using the Vaccine Adverse Event Reporting System, 2006-2017. Perspect Clin Res. 2020;11(1):24-30.
16. Ojha RP, Jackson BE, Tota JE, Offutt-Powell TN, Singh KP, Bae S. Guillain-Barre syndrome following quadrivalent human papillomavirus vaccination among vaccine-eligible individuals in the United States. Human Vaccines and Immunotherapeutics. 2014;10(1):2908-13.
17. Geier DA, Geier MR. A case–control study of quadrivalent human papillomavirus vaccine-associated autoimmune adverse events. Clinical Rheumatology. 2015;34(7):1225-31.

18. Frisch M, Besson A, Clemmensen KKB, Valentiner-Branth P, Molbak K, Hviid A. Quadrivalent human papillomavirus vaccination in boys and risk of autoimmune diseases, neurological diseases and venous thromboembolism. Int J Epidemiol. 2018;47(2):634-41.
19. Skufca J, Ollgren J, Artama M, Ruokokoski E, Nohynek H, Palmu AA. The association of adverse events with bivalent human papilloma virus vaccination: A nationwide register-based cohort study in Finland. Vaccine. 2018;36(39):5926-33.
20. Grönlund O, Herweijer E, Sundström K, Arnheim-Dahlström L. Incidence of new-onset autoimmune disease in girls and women with pre-existing autoimmune disease after quadrivalent human papillomavirus vaccination: a cohort study. Journal of Internal Medicine. 2016;280(6):618-26.
21. Hviid A, Svanström H, Scheller NM, Grönlund O, Pasternak B, Arnheim-Dahlström L. Human papillomavirus vaccination of adult women and risk of autoimmune and neurological diseases. Journal of Internal Medicine. 2018;283(2):154-65.
22. Grimaldi-Bensouda L, Guillemot D, Godeau B, Bénichou J, Lebrun-Frenay C, Papeix C, et al. Autoimmune disorders and quadrivalent human papillomavirus vaccination of young female subjects. Journal of Internal Medicine. 2014;275(4):398-408.
23. Grimaldi-Bensouda L, Rossignol M, Koné-Paut I, Krivitzky A, Lebrun-Frenay C, Clet J, et al. Risk of autoimmune diseases and human papilloma virus (HPV) vaccines: Six years of case-referent surveillance. Journal of Autoimmunity. 2017;79:84-90.
24. Miranda S, Chaignot C, Collin C, Dray-Spira R, Weill A, Zureik M. Human papillomavirus vaccination and risk of autoimmune diseases: A large cohort study of over 2 million young girls in France. Vaccine. 2017;35(36):4761-8.
25. Andrews N, Stowe J, Miller E. No increased risk of Guillain-Barré syndrome after human papilloma virus vaccine: A self-controlled case-series study in England. Vaccine. 2017;35(13):1729-32.
26. Cameron RL, Ahmed S, Pollock KGJ. Adverse event monitoring of the human papillomavirus vaccines in Scotland. Internal Medicine Journal. 2016;46(4):452-7.
27. Willame C, Rosillon D, Zima J, Angelo MG, Stuurman AL, Vroling H, et al. Risk of new onset autoimmune disease in 9- to 25-year-old women exposed to human papillomavirus-16/18 AS04-adjuvanted vaccine in the United Kingdom. Human Vaccines and Immunotherapeutics. 2016;12(11):2862-71.
